# Micelle-Triggered β-Hairpin to α-Helix Transition in a 14-Residue Peptide from a Choline-Binding Repeat of the Pneumococcal Autolysin LytA

**DOI:** 10.1002/chem.201500447

**Published:** 2015-04-27

**Authors:** Héctor Zamora-Carreras, Beatriz Maestro, Erik Strandberg, Anne S Ulrich, Jesús M Sanz, M Ángeles Jiménez

**Affiliations:** [a] Instituto de Química Física Rocasolano (IQFR), Consejo Superior de Investigaciones Científicas (CSIC)Serrano 119, 28006-Madrid (Spain); [b] Instituto de Biología Molecular y Celular, Universidad Miguel HernándezElche, 03202-Alicante (Spain); [c] Institute of Biological Interfaces (IBG-2), Karlsruhe Institute of Technology (KIT)P.O.B. 3640, 76021 Karlsruhe (Germany); [d] Institute of Organic Chemistry, Karlsruhe Institute of Technology (KIT)Fritz-Haber-Weg 6, 76131 Karlsruhe (Germany)

**Keywords:** micelles, protein folding, protein structures, structural biology, structural elucidation

## Abstract

Choline-binding modules (CBMs) have a ββ-solenoid structure composed of choline-binding repeats (CBR), which consist of a β-hairpin followed by a short linker. To find minimal peptides that are able to maintain the CBR native structure and to evaluate their remaining choline-binding ability, we have analysed the third β-hairpin of the CBM from the pneumococcal LytA autolysin. Circular dichroism and NMR data reveal that this peptide forms a highly stable native-like β-hairpin both in aqueous solution and in the presence of trifluoroethanol, but, strikingly, the peptide structure is a stable amphipathic α-helix in both zwitterionic (dodecylphosphocholine) and anionic (sodium dodecylsulfate) detergent micelles, as well as in small unilamellar vesicles. This β-hairpin to α-helix conversion is reversible. Given that the β-hairpin and α-helix differ greatly in the distribution of hydrophobic and hydrophilic side chains, we propose that the amphipathicity is a requirement for a peptide structure to interact and to be stable in micelles or lipid vesicles. To our knowledge, this “chameleonic” behaviour is the only described case of a micelle-induced structural transition between two ordered peptide structures.

## Introduction

**Figure 1:**
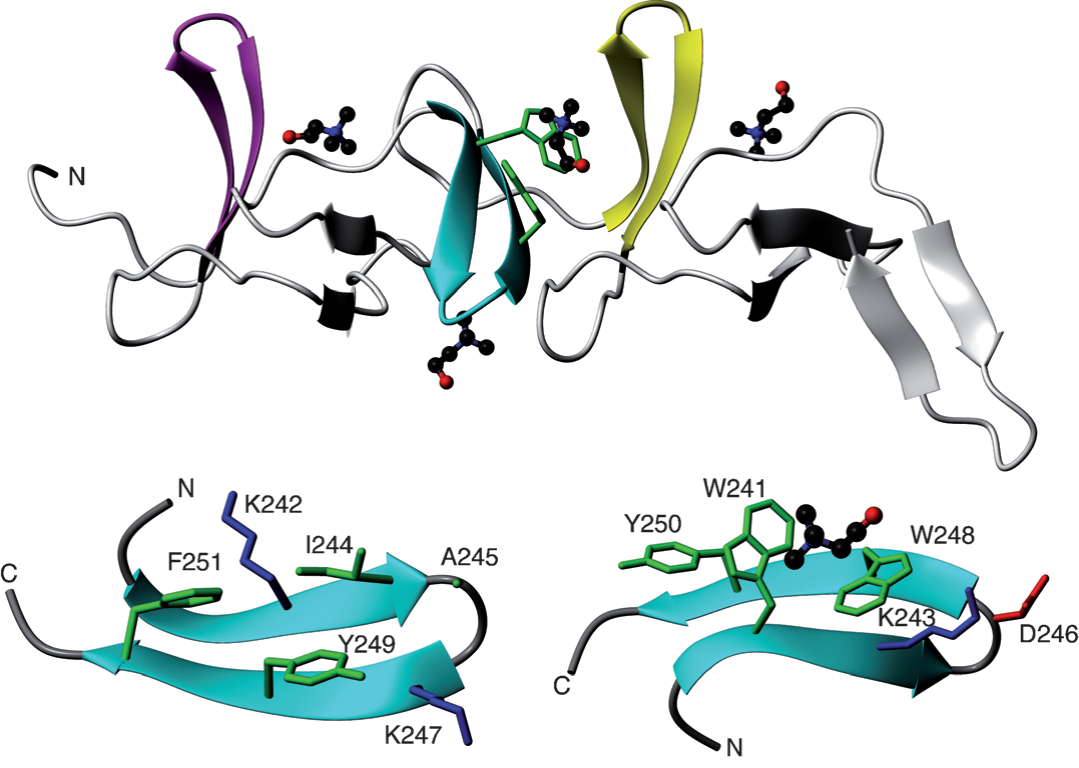
Top: Ribbon representation of the crystal structure of the choline-binding module of the LytA pneumococcal autolysin, C-LytA protein (PDB code: 1gvm). The three regular 2:2 β-hairpins are coloured: CBR1 in magenta, CBR3 in cyan and CBR4 in yellow. The bound choline molecules are displayed in ball-and-stick CPK-coloured representation. Trp side chains of the third β-hairpin are shown in green. Bottom: Two orientations of the CBR3 β-hairpin, residues 239–252. Each orientation displays the side chains of residues pointing out from the same β-sheet face. The positively charged residues are coloured blue, the negatively charged residues red, and aliphatic and aromatic green. The amino and carboxylate termini are labelled by “N” and “C”’, respectively.

Choline-binding proteins (CBPs) are a family of proteins that are present on the surface of several microorganisms, including pathogenic bacteria such as *Streptococcus pneumoniae* (pneumococcus), where they play an important role in the viability and virulence of the organism.^1^ These proteins display modular structures consisting of a catalytic domain that is responsible for the protein functionality, and a choline-binding module (CBM) that attaches the CBP to the cell surface through choline residues present in the teichoic and lipoteichoic acids.^2^ The choline-binding domains have a ββ-solenoid structure composed of approximately 20-residue choline-binding repeats (CBRs). Each standard CBR contains an approximately 14-residue β-hairpin followed by a six-residue linker sequence, with the choline molecule being bound between two consecutive repeats through hydrophobic and cation–π interactions with aromatic residues.^3^ In particular, the CBM from the LytA autolysin (C-LytA) consists of six repeats (CBR1-6) and four bound choline molecules, because the last hairpin is involved in protein dimerisation instead of ligand binding^4^ (Figure 1).

All these CBMs, and especially C-LytA, possess interesting biotechnological applications as affinity tags for protein immobilisation and purification by affinity chromatography,^5^ and are also good models that can be used to understand the folding and stability of repeat proteins.^6^ In this context, we aimed to find whether minimal peptides encompassing the sequence of a single CBR or even only its β-hairpin core maintains its native structure and the ability to bind choline. We previously studied a peptide derived from the β-hairpin core of the first repeat (LytA_197–210_; Figure 1)^7^ because the sequence of its loop is the statistically most suitable for a Type I′ β-turn.^8^ Our structural studies confirmed that peptide LytA_197–210_ forms a native-like β-hairpin structure in aqueous solution in an appreciable amount (63 % at 15 °C).^7^ Based on circular dichroism (CD) data, this peptide is also able to bind tetramethylamonium, a simple choline analogue. These promising results prompted us to look for a more robust structure by studying peptides encompassing the sequence of other C-LytA repeats. To this end, we compared the sequence and structural features of the CBRs of C-LytA. According to promotif analysis as reported in PDBsum (https://www.ebi.ac.uk/pdbsum/), the CBR1, CBR3 and CBR4 repeats are regular 2:2 β-hairpins with a Type I′ β-turn, the CBR2 is a 7:9 β-hairpin that has a very long turn region, the CBR5 is a 4:6 β-hairpin, and the CBR6 is a 3:5 β-hairpin. Excluding the already studied CBR1,^7^ and having in mind that the turn sequence is essential for β-hairpin folding and stability,^9^ and that 2:2 β-hairpins with Type I′ β-turns are particularly stable, the CBR3 and CBR4 repeats look to be the most promising to derive peptides that are able to maintain native structures independently. Based on the theoretical pI values (9.4 for CBR3, and 5.6 for CBR4), we considered that CBR3 should be more soluble in the pH range suitable for NMR studies (pH<7). Hence, we selected the β-hairpin sequence of CBR3 (peptide LytA_239–252_, TGWKKIADKWYYFN; Figure 1), and undertook its structural characterization under different solvent conditions, both in aqueous solution and in the presence of detergent micelles. It should be pointed out here that complete LytA amidase hydrolyses the cell wall peptidoglycan, causing high levels of autolysis of pneumococcal cells at the end of the stationary phase in liquid cultures.^10^ The biological significance of autolysis is still a matter of debate, but it may be related to important events in pneumococcal virulence because cell lysis releases toxins that may help bacterial dissemination in the infected individual, as well as DNA that may be used to transform other pneumococcal cells.^11^ The mechanism by which the protein LytA and other CBPs translocate from the cytoplasm to the cell wall without a peptide signal is still unknown, but it may involve the interaction with cell membranes, so that the structural study in micelles could shed some light on these kinds of events.

## Results

### Circular dichroism experiments

**Figure 2:**
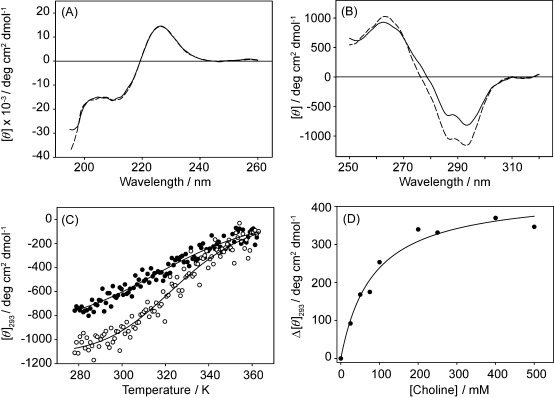
Spectroscopic characterization of LytA_239–252_ by circular dichroism: A) Far-UV CD spectrum in 20 mm Gly buffer, pH 3.0 at 25 °C, in the absence (solid line) and the presence (dashed line) of 500 mm choline; B) Near-UV CD spectrum. Line pattern as in A); C) Thermal unfolding of LytA_239–252_ in the absence (filled circles) and the presence (empty circles) of 500 mm choline. Solid lines represent the fits to the Gibbs–Helmholtz equation [Eq. (1)]; D) CD-monitored titration of LytA_239–252_ with choline (Δ[*θ*]_293_=[*θ*]${{{{\rm no}\>{\rm choline}\hfill \atop 293\hfill}}}
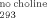
−[*θ*]${{{{\rm choline}\hfill \atop 293\hfill}}}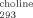). Data were fitted to a simple binding model [Eq. (3)].

Far and near-UV CD spectra (Figure 2 A and B) were recorded for LytA_239–252_ in aqueous solution at pH 3.0 (20 mm glycine buffer). The strong positive band at 227 nm observed in the far-UV spectrum, together with the significant near-UV CD signal, resemble those observed for the full-length C-LytA6a, 12 that have been described as arising from aromatic rings in rigid conformations. Therefore, this result suggests that peptide LytA_239–252_ forms a well-ordered, native-like structure in aqueous solution. On the other hand, the ability of LytA_239–252_ to bind choline was also examined by CD analysis. As observed in Figure 2 B, the near-UV bands at 293 nm (attributable to Trp side chain) and 286 nm (Tyr and Trp) become more intense in the presence of the ligand (500 mm). This suggests that the peptide is able to bind choline even in the absence of the following CBR4. However, somewhat strikingly, the far-UV CD spectrum is unaffected by the presence of choline (Figure 2 A), contrary to the full length C-LytA6a, 12 and the LytA_197–210_ peptide corresponding to the first hairpin.^7^ The lack of change in the far-UV CD spectrum (Figure 2 A) can be explained by the fact that, as demonstrated by NMR (see below), LytA_239–252_ has already acquired the whole of the secondary structure in the absence of ligand, meanwhile both the free C-LytA module6a and the LytA_197–210_ repeat^7^ are only partially folded in solution and need choline additionally to fully acquire structure.

We also examined the thermal stability of LytA_239–252_ by monitoring the temperature dependence of the molar ellipticity at 293 nm in the range 5–90 °C, both in the absence and in the presence of 500 mm choline (Figure 2 C). In the two cases, heating led to featureless CD spectra, indicative of a massive loss of structure, which was reversible upon cooling (see the Supporting Information, Figure S1). We first fitted the transition data to the Gibbs–Helmholtz equation [see Experimental Section, Eq. (1)] assuming an approximated Δ*C*_p_ of 1.6 kJ mol^−1^, the value reported for the 12-residue, tryptophan zipper, trpzip4 hairpin.^13^ However, data fitting using this parameter was very poor (data not shown). Therefore, because the thermal transitions show very little cooperativity, as expected from the lack of sizeable hydrophobic cores and tight packing around the aromatic residues (see below), we assumed the contribution of Δ*C_p_* to be negligible, which is an approximation already followed for other β-hairpin peptides.^14^ The thermodynamic parameters of LytA_239–252_ calculated by using this approach are Δ*H*_m_=38±4 kJ mol^−1^, *T*_m_=321±2 K (48 °C) and Δ*G* (25 °C)=2.5±0.4 kJ mol^−1^ for the peptide in glycine buffer at pH 3.0. The stability at 25 °C is intermediate between that of the 15-residue SESYW11 hairpin (0.1 kJ mol^−1^),14a and the tryptophan zippers trpzip3 (4.6 kJ mol^−1^) and trpzip4 (6.3 kJ mol^−1^), although in the latter cases the Trp/Trp stacking contributes decisively to stability.^13^ In the presence of 500 mm choline, the results were Δ*H*_m_=50±4 kJ mol^−1^, *T*_m_=326±1 K (53 °C) and the stability increased to Δ*G* (25 °C)=4.2±0.4 kJ mol^−1^. It is noteworthy that the two unfolding traces converge at around 70 °C (Figure 2 C), indicating that the peptide is competent to bind choline up to these high temperatures.

The affinity of LytA_239–252_ for choline was calculated by recording near-UV CD spectra at 25 °C and different ligand concentrations. The plot of the change in molar ellipticity at 293 nm against choline concentration was fitted to Equation (3) [see Experimental Section], assuming one binding site (Figure 2 D), so that the dissociation constant (*K*_d_) was calculated as 80±10 mm.

### LytA_239–252_ acquires a native-like β-hairpin in aqueous solution

**Figure 3:**
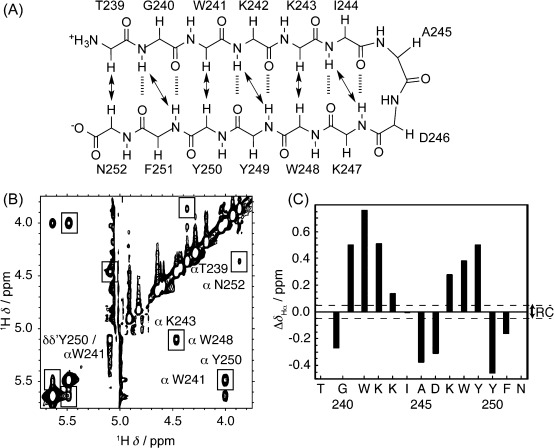
NMR data for LytA_239–252_ in aqueous solution: A) Schematic representation of the 2:2 β-hairpin formed by LytA_239–252_ in aqueous solution. Double arrows indicate the NOEs observed in 2D NOESY spectra. B) 2D NOESY spectra of LytA_239–252_ in D_2_O at pH 3.0 and 5 °C. Nonsequential NOEs are boxed and labelled at one of the diagonal sides. C) Bar plot of Δ*δ*_Hα_ (Δ*δ*_H*α*_=${\delta {{{\rm observed}\hfill \atop {\rm H}{\rm \alpha} \hfill}}}
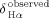
−${\delta {{{\rm RC}\hfill \atop {\rm H}{\rm \alpha} \hfill}}}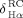, ppm) as a function of sequence for peptide LytA_239–252_ in H_2_O/D_2_O 9:1 v/v at pH 3.0 and 25 °C. ${\delta {{{\rm RC}\hfill \atop {\rm H}{\rm \alpha} \hfill}}}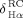 values were taken from Wishart et al.^17^ Values for the two Gly Hα protons are plotted. The N- and C-terminal residues are not shown. The dashed lines indicate the random coil (RC) range.

To determine the structure adopted by peptide LytA_239–252_ in aqueous solution, 1D and 2D NMR spectra were recorded of a 1 mm sample. The ^1^H and ^13^C chemical shifts were assigned by following a standard strategy (see Materials and Methods). The nonsequential NOE cross-peaks observed in 2D ^1^H,^1^H-NOESY spectra include those characteristic of antiparallel β-sheets (Figure 3 A and B); that is, those between the Hα protons of residues facing each other in non-hydrogen-bonded sites, and between amide protons of residues facing each other in hydrogen-bonded sites. The presence of these NOEs shows that peptide LytA_239–252_ in aqueous solution adopts a β-hairpin structure, and that the β-strand register is native-like. Formation of β-hairpin structures is further confirmed by the plot of Δ*δ*_Hα_, Δ*δ*_Cα_ and Δ*δ*_Cβ_ as a function of peptide sequence; that is, two stretches of positive Δ*δ*_Hα_ and Δ*δ*_Cβ_ values, and negative Δ*δ*_Cα_ corresponding to residues at N- and C-terminal strands, which are separated by Δ*δ*_Hα_, Δ*δ*_Cα_ and Δ*δ*_Cβ_ values of the corresponding opposite sign at the turn region14a, 15 (Figure 3 C and S2 in the Supporting Information). Based on the averaged Δ*δ*_Hα_ values at the strand residues (+0.42 ppm at 25 °C) and considering that the averaged Δ*δ*_Hα_ value at protein β-strands is +0.40 ppm,^16^ the β-hairpin population formed in aqueous solution at pH 3.0 and 25 °C is approximately 100 %. This demonstrates that LytA_239–252_ is a more robust hairpin than the previously studied LytA_190–210_ peptide (63 % β-hairpin population in aqueous solution at 15 °C).^7^

**Figure 4:**
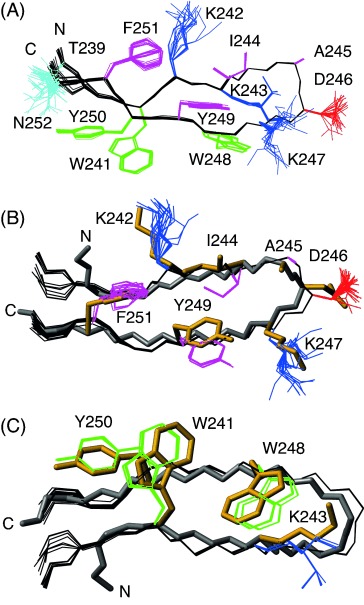
Structure of LytA_239–252_ in aqueous solution: A) Ensemble of the 20 lowest target function structures overlaid onto the backbone atoms (black). Side chains are coloured in blue if positively charged, in red if negatively charged, and in cyan if polar. Aromatic and hydrophobic side chains are in magenta if pointing upwards and in green if pointing downwards. B) and C) Backbone atoms of LytA_239–252_ (in black) overlaid onto the corresponding atoms in the crystalline C-LytA structure (in grey neon; 1gvm). Side chains of residues at the turn region and at hydrogen-bonded sites are shown in B) and those of residues at non-hydrogen-bonded sites in C). LytA_239–252_ side chains are coloured as in A), and those of C-LytA in gold neon. The amino and carboxylate termini are labelled by “N” and “C”, respectively.

To obtain further details of the features of this structure, a structure calculation was performed on the basis of the distance restraints derived from the complete set of observed NOEs and the dihedral angle restraints obtained from the ^1^Hα, ^13^Cα and ^13^Cβ chemical shifts by using the program TALOS+ (Table 1 and Table S2). The calculated structure (Figure 4 A) is well defined, as indicated by the small pairwise RMSD presented by the backbone atoms, 0.3±0.1 Å (Table 1), and is very similar to the native structure, as can be appreciated in Figure 4 B and C. The side chains for all the residues, except for the N- and C-terminal, are also ordered, because their χ1 dihedral angles show very little variation among the 20 calculated structures (Table S2), and display orientations quite similar to those in the native protein (Figure 4 B and C).

**Table 1:** Main structural statistics parameters for the ensemble of the 20 lowest target function conformers calculated for peptide LytA_239–252_ in aqueous solution and in micelles.

	Aqueous solution	DPC micelles	SDS micelles
**total number of restraints**			
upper limit distance	172	156	237
*φ* and *ψ* dihedral angle	24	24	23
			
**pairwise RMSD [Å]**			
backbone atoms	0.3±0.1	0.6±0.2	0.2±0.1
all heavy atoms	1.0±0.1	1.4±0.4	0.9±0.2

### LytA_239–252_ undergoes a reversible β-hairpin to α-helix transition induced by DPC micelles

**Figure 5:**
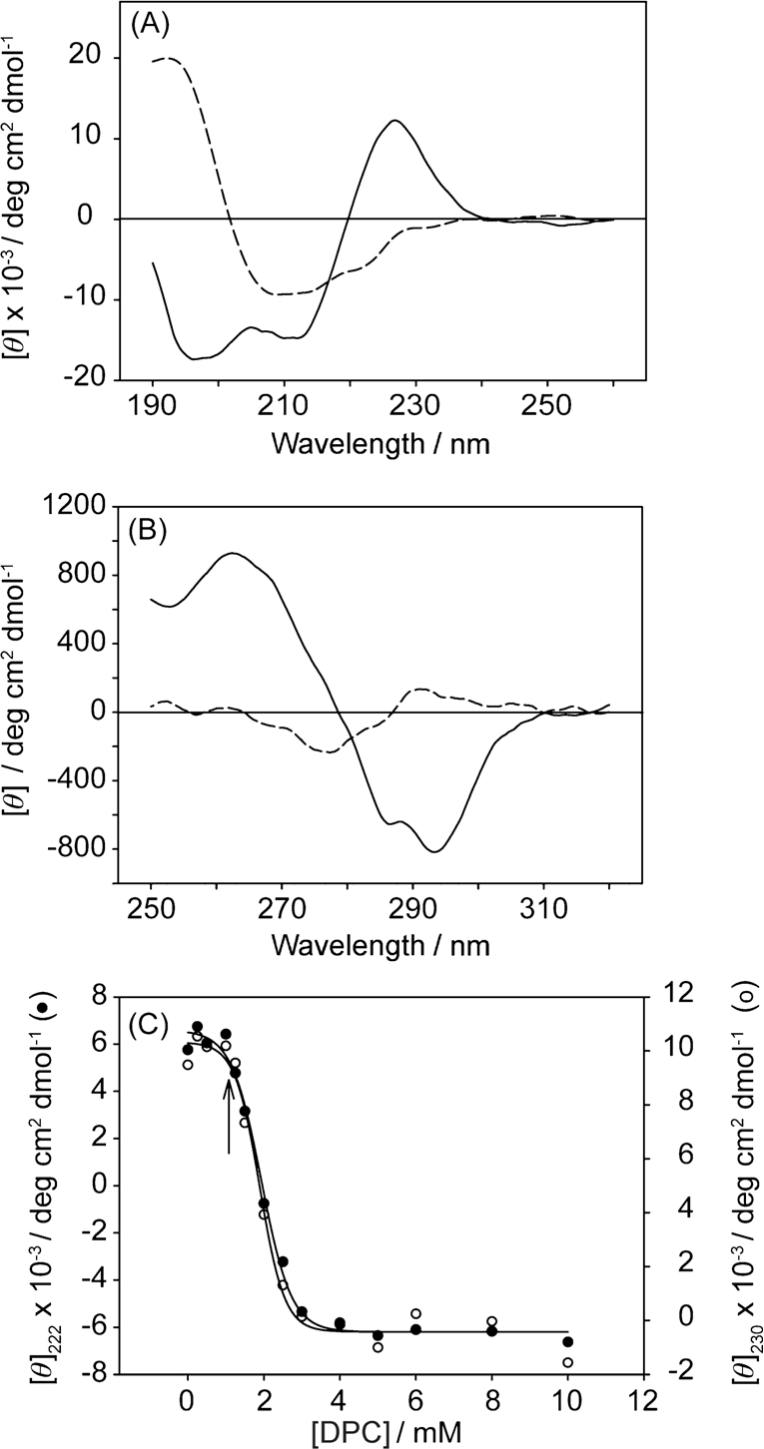
Spectroscopic characterisation of LytA_239–252_ in the presence of DPC: A) Far-UV and B) near-UV CD spectra in 20 mm Gly buffer, pH 3.0, at 25 °C in the absence (solid line) and the presence (dashed line) of 30 mm DPC; C) Titration of LytA_239–252_ with DPC monitoring the ellipticity at 222 (filled circles) and 230 nm (open circles). The arrow indicates the critical micelle concentration (cmc) of DPC.

In an attempt to check whether regions of LytA could interact with the cell membrane to translocate from the cytoplasm to the cell wall without a signal peptide, we studied the structure of LytA_239–252_ in the presence of DPC, because micelles of this compound represents a simple membrane model, commonly used for solution NMR studies.^18^ First, we recorded the far-UV CD spectrum for the peptide LytA_239–252_ in the presence of 30 mm DPC (peptide/detergent ratio 1:30) (Figure 5 A). Addition of detergent clearly changes the aromatic-dominated spectrum of the peptide to a broad, negative band with a minimum at 209 nm and a shoulder at 222 nm, characteristic of α-helices with some contribution of β-structures.^19^ The loss of anisotropic environment around the aromatic side chains is also evident in the near-UV CD spectrum (Figure 5 B), which only displays a small minimum at 276 nm and a maximum at 290 nm of small magnitude. A titration with increasing concentrations of DPC is shown in Figure 5 C. Transition from the β-hairpin to the α-helix occurs cooperatively and independently of the monitored wavelength. Moreover, the CD spectral transition occurs with an isodichroic point at 217 nm (see the Supporting Information, Figure S3 A). These two facts suggest that the structural conversion takes place between two states and without any detectable intermediates. When subjected to a thermal scan, the helical structure accumulated in 30 mm DPC is gradually lost in a non-cooperative way (Figure S3B, inset), indicating the lack of a detectable hydrophobic core, although unfolding was reversible upon cooling (Figure S3B). On the other hand, to determine whether DPC monomers or micelles are responsible for the structural transition in LytA_239–252_, we determined the critical micelle concentration (cmc) of DPC under our experimental conditions (20 mm glycine buffer, pH 3.0), which was estimated as 1.2 mm (Figure S3C). As depicted in Figure 5 C, this concentration is right at the onset of the cooperative transition. Therefore, we can hypothesise that interaction of LytA_239–252_ with DPC micelles drives the conformational change of the peptide to a largely helical structure.

**Figure 6:**
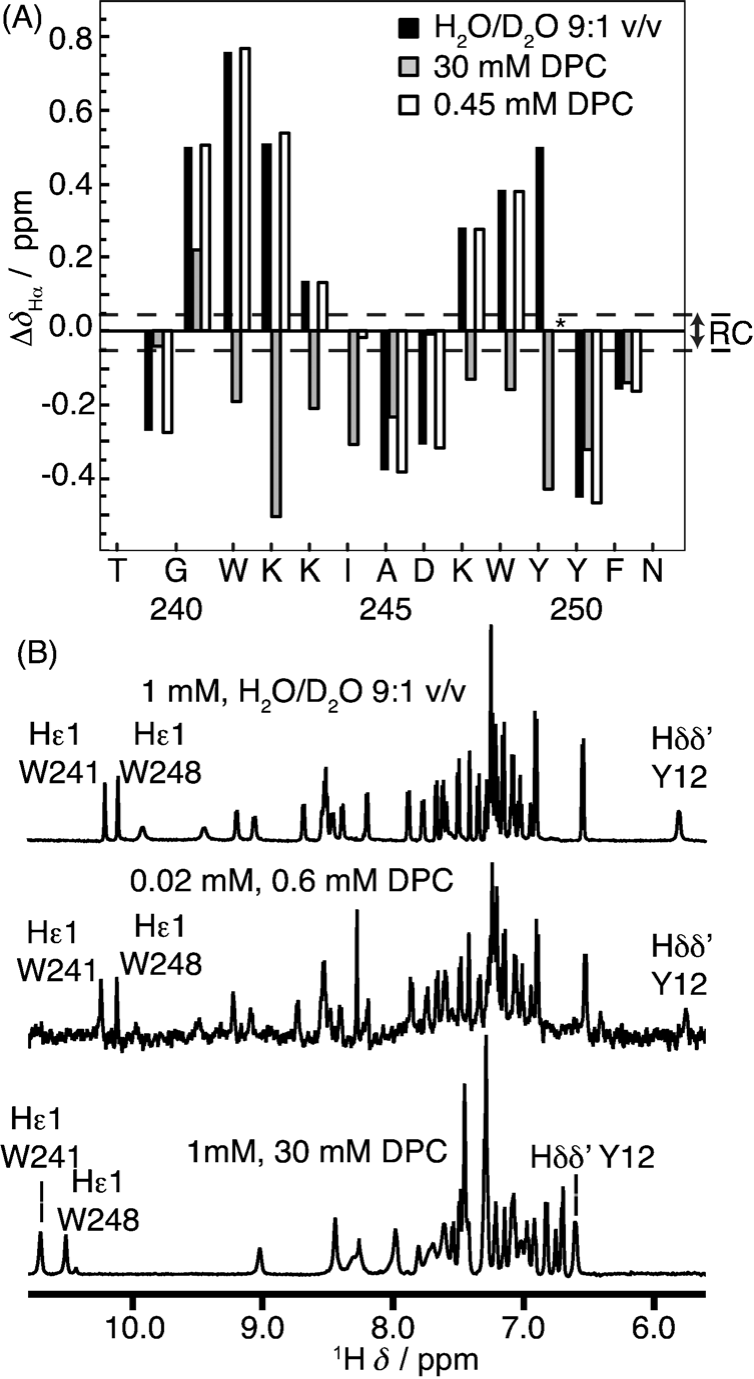
NMR data for LytA_239–252_ in the presence of DPC: A) Plots of Δ*δ*_Hα_ (Δ*δ*_H*α*_=${\delta {{{\rm observed}\hfill \atop {\rm H}{\rm \alpha} \hfill}}}
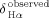
−${\delta {{{\rm RC}\hfill \atop {\rm H}{\rm \alpha} \hfill}}}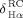, ppm) as a function of peptide sequence for LytA_239–252_ in H_2_O/D_2_O 9:1 v/v (black bars), in 30 mm [D_38_]DPC (grey bars) and in 0.45 mm [D_38_]DPC (white bars) at pH 3.0 and 25 °C. ${\delta {{{\rm RC}\hfill \atop {\rm H}{\rm \alpha} \hfill}}}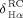 values were taken from Wishart et al.^17^ The N- and C-terminal residues are not shown. The dashed lines indicate the random coil (RC) range, and the asterisks indicate that the corresponding *δ*_Hα_ values were not determined. B) Selected regions of the 1D ^1^H NMR spectra of LytA_239–252_ at pH 3.0 and 25 °C at 1 mm concentration in 30 mm [D_38_]DPC in H_2_O/D_2_O 9:1 v/v (bottom), at 0.02 mm concentration in 0.6 mm [D_38_]DPC in H_2_O/D_2_O 9:1 v/v (middle), and at 1 mm concentration in H_2_O/D_2_O 9:1 v/v (top). Peptide/detergent ratio in DPC-containing samples is 1:30.

We then proceeded to characterise LytA_239–252_ in the presence of DPC by NMR spectroscopic analysis. First, we acquired 1D and 2D NMR spectra of the peptide in the presence of 0.5 mm DPC, a concentration below the cmc. Under these conditions, the NMR spectra are essentially identical to those in aqueous solution (see 2D ^1^H,^13^C-HSQC spectra, Figure S4 A in the Supporting Information). The profile of conformational shifts (Figure 6 A) provides further confirmation that peptide LytA_239–252_ in 0.5 mm DPC adopts a β-hairpin structure. On the other hand, as occurs in the case of CD spectra, NMR spectra of peptide LytA_239–252_ in 30 mm DPC are very different from those in aqueous solution, as observed in the 1D ^1^H NMR spectra shown in Figure 6 B (see also 2D ^1^H,^13^C-HSQC spectra, Figure S4B in the Supporting Information). The observed differences look larger than would be expected to be observed based on the effect of solvent on chemical shifts. Indeed, the profiles of conformational shifts in the presence of DPC micelles are completely different from those in aqueous solution (Figure 6 A). It is noticeable that those residues with positive Δ*δ*_Hα_ values in aqueous solution have negative values in the presence of DPC micelles. Instead of a profile characteristic of β-hairpin structures, as observed in aqueous solution, the profile observed in the presence of DPC micelles is that typical of helices; that is, negative Δ*δ*_Hα_ and Δ*δ*_Cβ_ values, and positive Δ*δ*_Cα_ values for residues 241–251. Further confirmation about the formation of a helix in DPC micelles comes from the set of NOEs, which include those characteristic of helices; i.e., αN_(*i*,*i*+3)_, αβ_(*i*,*i*+3)_ and strong sequential NN_(*i*,*i*+1)_ (see the Supporting Information, Figure S5). This result, in accordance with the CD data, confirms that the formation of helix in peptide LytA_239–252_ is induced by DPC micelles. However, the peptide/detergent ratio was different at 0.5 mm DPC (ca. 1:1) than at 30 mm DPC (1:30 or 1:60). Therefore, as an additional check that the conformational change occurs in the presence of DPC micelles and not by interaction with the DPC monomer, we acquired a 1D ^1^H NMR spectrum at a peptide/detergent ratio of 1:30, but at a sub-micellar DPC concentration (0.6 mm DPC and 0.02 mm LytA_239–252_). As seen in Figure 6 B, the 1D ^1^H NMR spectra acquired under these conditions is essentially identical to that recorded in aqueous solution, except for the signal-to-noise ratio. This sample was prepared by a 1:50 dilution in water of an aliquot of a 1 mm LytA_239–252_ sample in 30 mm DPC, pH 3.0, in which the peptide forms a α-helix. Hence, the fact that, once diluted to a sub-micellar DPC concentration, its 1D ^1^H NMR spectra is identical to that of LytA_239–252_ in aqueous solution, in which the peptide forms a β-hairpin structure, provides evidence for the reversibility of the α-helix to β-hairpin transition, and confirms the role of DPC micelles on the peptide conformational change.

**Figure 7:**
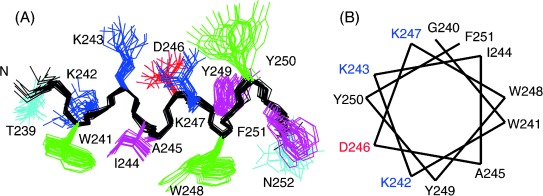
Structure of LytA_239–252_ in DPC micelles. A) Ensemble of the 20 lowest target function structures overlaid onto the backbone atoms (black). Side chains are coloured as in Figure 4. “N” indicates the amino end. B) Helical wheel representation of the side chain distribution. Positively charged residues are in blue, negatively charged in red, and aromatic and hydrophobic in black.

Considering that the population of the helix form estimated from the magnitude of the Δ*δ*_Hα_ values and the averaged Δ*δ*_Hα_ value at protein α-helices (−0.39 ppm)^20^ is quite high (62 % at 35 °C), we proceeded to calculate the peptide structure under these conditions. The resulting structure is depicted in Figure 7 A. The helical backbone is well defined, as well as most side chains, which exhibit small ranges of variation for the χ1 and χ2 dihedral angles in most residues (Table 1). The packing of side chains in this α-helix structure (Figure 7 A) and in the β-hairpin formed in aqueous solution (Figure 4 A) is very different. For example, the pairs W241/Y250 and I244/Y249 are close in the β-hairpin structure, but far away in the α-helix (Figure 4 A and Figure 7 A).

### How does LytA_239–252_ interact with DPC micelles?

DPC micelles are relatively spherical bodies with a radius of approximately 18.6–23.3 Å, formed by 44–61 monomers per micelle.^21^ The length of the helix formed by LytA_239–252_ in the presence of DPC micelles, which was measured from the N to C-end distances in the calculated structures by using MOLMOL,^22^ is approximately 22 Å. The peptide helix could, in principle, be lying on the micelle surface, or be immersed either totally or partially into the micelles, although the former hypothesis is supported by the helical-wheel analysis shown in Figure 7 B that clearly depicts an amphipathic helix with hydrophobic and polar faces noticeably segregated.

**Figure 8:**
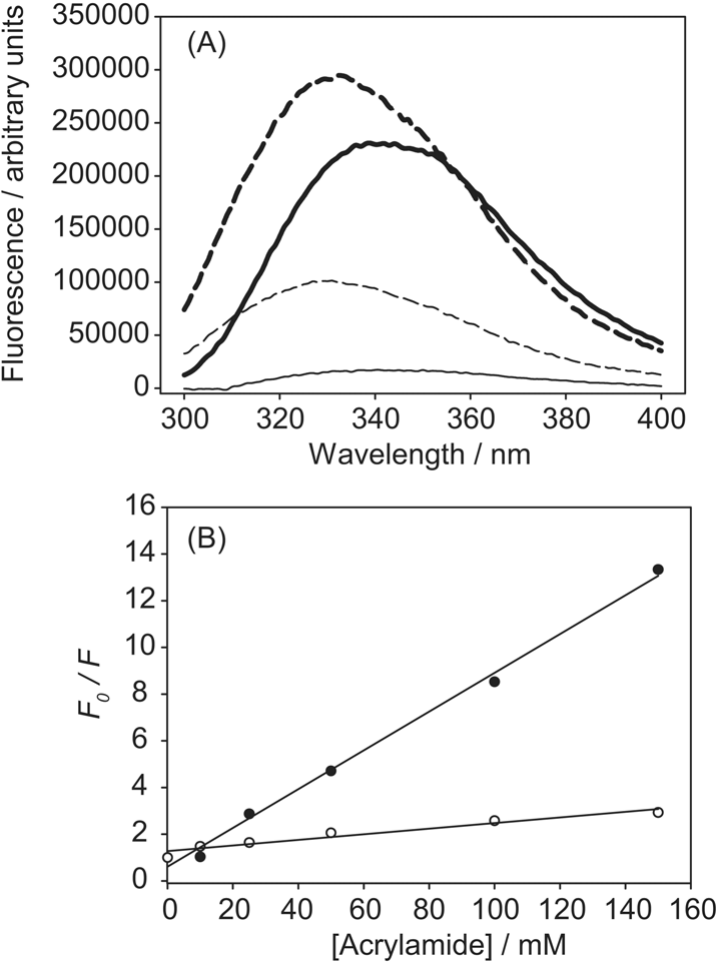
Intrinsic fluorescence of LytA_239–252_: A) Wavelength spectra in 20 mm Gly buffer, pH 3.0 and 25 °C, upon excitation at 280 nm. Thick solid line, no addition; thin solid line, after addition of 150 mm acrylamide; thick dashed line, after addition of 30 mm DPC; thin dashed line, after addition of 30 mm DPC plus 150 mm acrylamide; B) Stern–Volmer plot for acrylamide quenching in the absence (filled circles) and the presence (empty circles) of DPC micelles [Eq. (4)]. *F*_0_ and *F* represent the fluorescence intensity at 340 nm in the absence and the presence of the quencher, respectively.

To gain information on the environment around the aromatic residues in the presence of DPC micelles, the intrinsic fluorescence spectra of the peptide upon excitation at 280 nm were recorded (Figure 8 A). In the absence of detergent, the emission spectrum is dominated by tryptophan contributions, with a maximum at 340 nm, indicating a high solvent exposure. Addition of DPC micelles caused a blueshift in the spectrum maximum to 331 nm concomitant with an increase in fluorescence intensity. This indicates that the Trp residues are in a less polar environment and more buried from solvent in the presence of DPC micelles than in aqueous solution. Moreover, we carried out acrylamide quenching experiments in the absence and presence of detergent. Figure 8 A shows that the quencher affects the Trp fluorescence to a much higher extent in aqueous solution than in the presence of DPC. Stern–Volmer analysis of the data (Figure 8 B) yields quenching constants *K*_SV_ (no DPC)=80±3 m^−1^ and *K*_SV_ (DPC micelles)=10±1 m^−1^. This result indicates that there is a physical impediment for the quencher to reach the Trp residues when DPC micelles are formed. These differences cannot simply arise from the Trp side chains being less accessible in the helical conformation than in the β-hairpin, because the solvent-accessible areas of these residues in the two structures, calculated using MOLMOL,^22^ are quite similar: 47 % for W241 and 43 % for W248 in the β-hairpin (Figure 4 A), and 46 % for W241 and 47 % for W248 in the α-helix (Figure 7 A). Therefore, the fluorescence data suggest that the Trp side chains located in the hydrophobic face of the helix (Figure 7 B) are immersed in the micelle. In fact, in contrast to most polar side chains, the indole rings are very ordered in the helix formed by LytA_239–252_ in DPC (Figure 7 A), indicating a rigid environment that restricts their fluctuation.

To further corroborate the conclusion that the Trp indole rings interact with the DPC aliphatic chain and to better characterise how the peptide helix interacts with the micelle, we re-examined the 2D NOESY spectra of LytA_239–252_ in 30 mm [D_38_]DPC to search for intermolecular NOEs. Notably, although deuterated [D_38_]DPC was used, the degree of deuteration was only 98 % (see Materials and Methods), so that the averaged concentration of non-deuterated DPC in a 30 mm [D_38_]DPC solution is 0.6 mm, a concentration that is sufficient to allow detection of the DPC NMR signals. In fact, DPC signals are observed in the 2D NMR spectra recorded for LytA_239–252_ in 30 mm [D_38_]DPC. Unfortunately, we could not see any intermolecular NOE, probably because of the dynamic nature of the DPC micelles themselves, because a micelle/monomer equilibrium is always present in solution, and also because of the peptide/micelle complex, which is in equilibrium with the unbound peptide and micelles.

We then examined the effect of hydro-soluble and lipo- soluble relaxation agents on the NMR signals of LytA_239–252_ in 30 mm [D_38_]DPC. Hydro-soluble paramagnetic compounds should affect the signals corresponding to residues lying outside the micelle, whereas lipo-soluble agents would affect those of residues buried inside the micelle.18b Upon titration with the hydro-soluble MnCl_2_, we observed that the α-NH cross-peaks of residues at the N-terminal moiety decrease in intensity, but remain visible in the 2D ^1^H,^1^H-TOCSY spectrum, whereas those of residues Y249–N252 at the C-terminal half disappear (see the Supporting Information, Figure S6 A). This suggests that this peptide segment either lies outside or points outwards from the micelle. In the case of lipo-soluble methyl-16-doxyl-stearate (free radical), which is a probe for the micelle centre, the α-NH cross-peaks that remain observable at the 2D ^1^H,^1^H-TOCSY spectrum are G240, K243, I244 (very weak), K247 and N252 (Figure S6B). These same α-NH cross-peaks plus those of Y250 and F251, although very weak (Figure S6C), persist upon titration with the lipo-soluble 5-doxyl-steararic acid (free radical). These persistent signals should correspond to residues outside the micelle or close to the surface of the micelle. Interestingly, the side chains for most of these residues are located at the same side of the α-helix (Figure 7). Nevertheless, the distinction between residues inside and outside the micelle is not accurate because of the dynamic character of the peptide/micelle complex (see above), and so some signals are mostly unaffected by both hydro- and lipo-soluble compounds ((see the Supporting Information, Figure S6).

On the whole, a picture that would fit both with fluorescence data and with the effect of paramagnetic compounds on NMR spectra is that the LytA_239–252_ helix lies in a slightly tilted position relative to the micelle diameter, probably quite close to the micelle surface, and the N-terminus holds most interactions with the micelle (see the Supporting Information, Figure S7). Furthermore, the hydrophobic face of the helix, which contains the Trp side chains, points towards the micelle centre, and the hydrophilic side, where Lys243 and Lys247 are placed, points to the micelle surface.

### DPC micelles do not induce helix formation in other β-hairpin peptides

**Figure 9:**
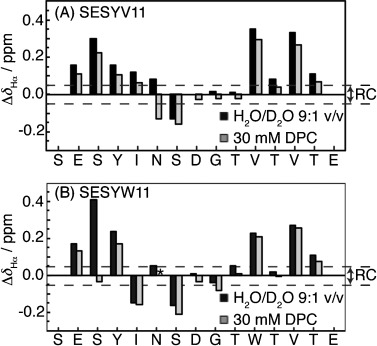
Bar plots of Δ*δ*_Hα_ (Δ*δ*_H*α*_=${\delta {{{\rm observed}\hfill \atop {\rm H}{\rm \alpha} \hfill}}}
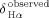
−${\delta {{{\rm RC}\hfill \atop {\rm H}{\rm \alpha} \hfill}}}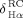, ppm) as a function of sequence for the β-hairpin peptides SESYV11 (A) and SESYW11 (B) in H_2_O/D_2_O 9:1 v/v (in black; data taken from Santiveri et al.,15a,28b,14a, 28a) and in 30 mm [D_38_]DPC (in grey). ${\delta {{{\rm RC}\hfill \atop {\rm H}{\rm \alpha} \hfill}}}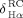 values are taken from Wishart et al.^17^ The N- and C-terminal residues are not shown. The dashed lines indicate the random coil (RC) range, and the asterisk indicates that the corresponding *δ*_Hα_ value was not determined.

Most of the structures determined so far in the presence of DPC micelles correspond to cationic antimicrobial peptides and cell-penetrating peptides, and can be structurally classified as 1) α-helical-prone peptides, which are mainly unstructured in aqueous solution and become helical in the presence of the micelles;18b 2) disulfide-rich peptides, some of which exhibit β-hairpin structures that are stabilised by one or more cross-strand disulfide bonds,^23^ and 3) Trp-rich peptides with complex structural behaviours, such as indolicidin^24^ and puroindoline derivatives.^25^ None of these groups include linear peptides adopting β-hairpin structures in DPC micellar media. In fact, as far as we know, only a linear octapeptide that adopts a β-hairpin structure in micelles has been reported,^26^ although this peptide contains a DPro-Gly turn sequence, which is known to nucleate β-hairpin structures,9b, 27 and also a myristoyl N-terminal extension. Therefore, to discard the possibility that the conformational change triggered by DPC micelles in peptide LytA_239–252_ is a consequence of a general nonspecific helix-inducer effect of DPC micelles, we examined whether other unrelated linear peptides that are able to form stable β-hairpins in aqueous solution become helical in DPC micelles. To this end, we selected two of our previously reported β-hairpin-forming peptides, SESYV11 and SESYW11,14a, 28 to be studied by NMR in 30 mm [D_38_]DPC. The profiles of Δ*δ*_Hα_, Δ*δ*_Cα_, and Δ*δ*_Cβ_ values (Figure 9 and Figure S8 in the Supporting Information) are quite similar to those in aqueous solution, indicating that the two peptides maintain their β-hairpin structures in the DPC micellar media, hence, confirming that the DPC micelles do not have a nonspecific helix-inducer effect.

### The β-hairpin structure formed by LytA_239–252_ remains in the presence of TFE

**Figure 10:**
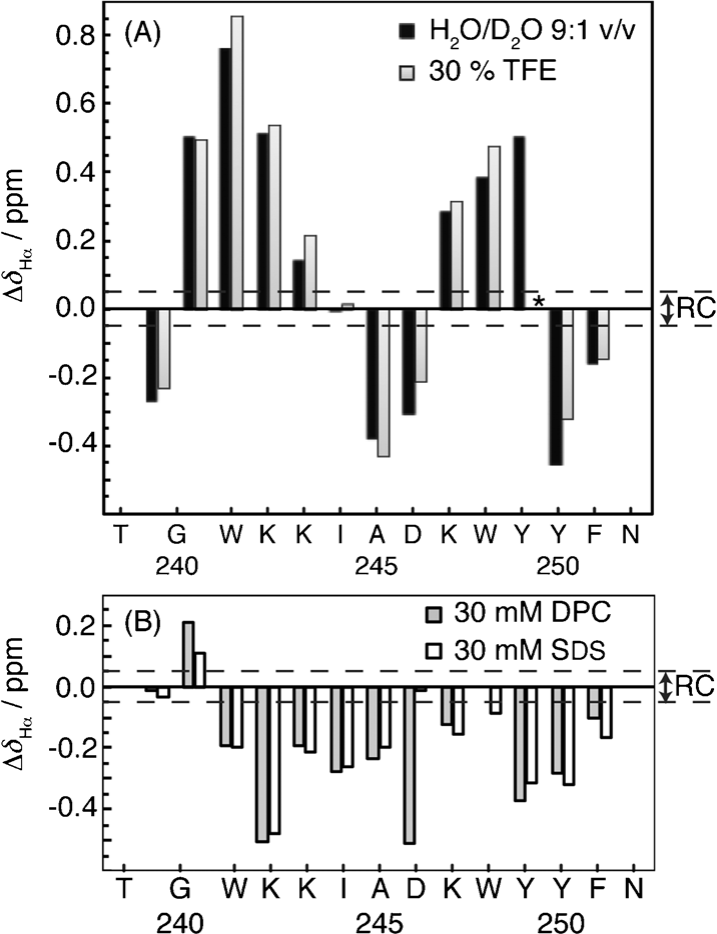
Bar plots of Δ*δ*_Hα_ (Δ*δ*_H*α*_=${\delta {{{\rm observed}\hfill \atop {\rm H}{\rm \alpha} \hfill}}}
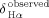
−${\delta {{{\rm RC}\hfill \atop {\rm H}{\rm \alpha} \hfill}}}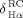, ppm) as a function of sequence for peptide LytA_239–252_ in different solvent conditions at pH 3.0 and 25 °C: A) in aqueous solution (black) and in 30 % TFE (grey), and B) in 30 mm DPC (grey) and in 30 mm SDS (white). ${\delta {{{\rm RC}\hfill \atop {\rm H}{\rm \alpha} \hfill}}}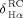 values were taken from Wishart et al.^17^ The N- and C-terminal residues are not shown. The dashed line indicates the range of random coil Δ*δ*_Hα_ values, and the asterisk indicates that the corresponding *δ*_Hα_ value was not determined.

TFE has been shown to stabilise β-hairpins,^29^ but it is most commonly known as a helix-inducer solvent.^30^ Given that LytA_239–252_ spontaneously adopts a native-like β-hairpin in aqueous solution, and an α-helix structure in DPC micelles, we were intrigued to know which of these structures the peptide would acquire in the presence of TFE. Thus, we proceeded to record 1D and 2D NMR spectra of LytA_239–252_ in 30 % TFE and assigned their ^1^H and ^13^C resonances. The Δ*δ*_Hα_, Δ*δ*_Cα_ and Δ*δ*_Cβ_ values plotted as a function of sequence (Figure 10 A and the Supporting Information S2) follow the same pattern as that in aqueous solution, which indicates that merely inducing intramolecular H-bonding is not sufficient to convert the LytA_239–252_ β-hairpin into an α-helix, and that an anisotropic environment such as that provided by detergent micelles is also necessary.

### LytA_239–252_ also forms a helix in negatively charged SDS micelles

Given that DPC contains a positively charged choline unit, the possibility existed that the quaternary amine could emulate the role of its counterpart in the cell-wall teichoic acids and, specifically, interact with the aromatic residues in the LytA_239–252_ choline-binding repeat. To check this hypothesis, we performed a structural NMR study of LytA_239–252_ in the presence of SDS, at concentrations both below (0.2 mm [D_25_]SDS) and above (30 mm [D_25_]SDS) cmc, for which reported values are in the range 1–8 mm.^18^ DPC and SDS have aliphatic chains of the same length (12-carbon atoms), but differ in their polar head-groups: zwitterionic in DPC and negatively charged in SDS. As in the case of DPC, the NMR spectra of LytA_239–252_ at sub-micellar SDS concentrations are similar to those in pure aqueous solution, whereas they completely differ in the presence of SDS micelles (see the Supporting Information, Figure S4D and S4E). Analogously, the profiles of Δ*δ*_Hα_, Δ*δ*_Cα_ and Δ*δ*_Cβ_ values in 30 mm SDS (Figure 10 B and Figure S2 in the Supporting Information) are very different to those observed in aqueous solution, and are very similar to those in 30 mm DPC; that is, they provide evidence that LytA_239–252_ in SDS micelles also adopts a helical structure. The set of non-sequential NOEs confirms the formation of a helix structure in SDS micelles, which has a population of 56 % at 25 °C, as estimated from the averaged Δ*δ*_Hα_ for residues 241–251.^20^ The structure in SDS micelles was calculated by following the same protocol as in aqueous solution and in DPC micelles (see Materials and Methods). The resulting α-helix is well defined (Table 1 and Figure S9 A in the Supporting Information) and is similar to that in DPC micelles (pairwise RMSD for backbone atoms of SDS versus DPC structures is 1.1±0.1 Å). This suggests that the influence of the choline head group in DPC is not relevant to inducing the hairpin to helix transition.

### LytA_239–252_ is also helical in lipid vesicles

**Figure 11:**
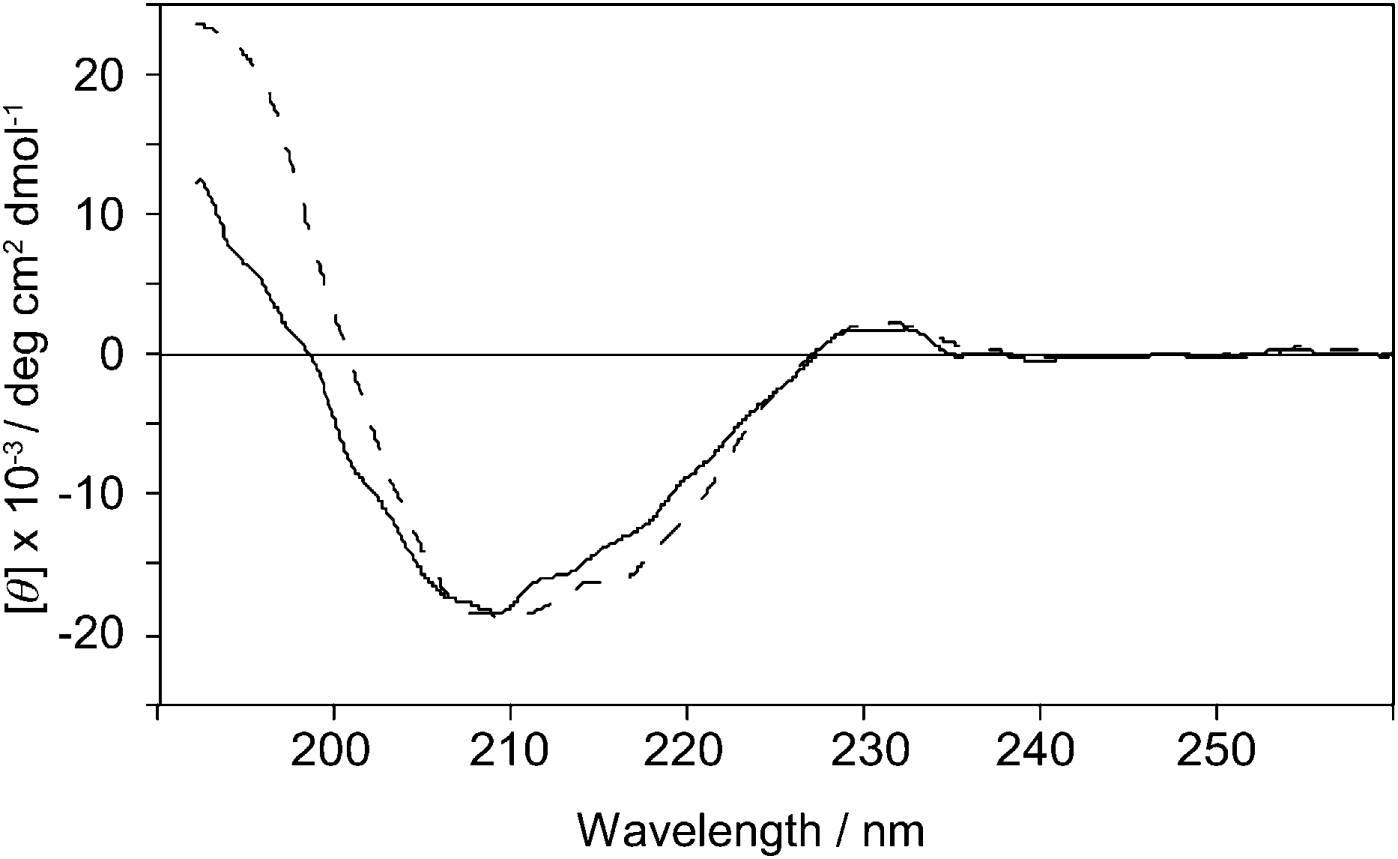
Far-UV CD spectra of LytA_239–252_ in vesicles: DMPC/DMPG (3:1) (red line) and POPE/POPG (2:1) (blue line). Both samples were prepared in 10 mm phosphate buffer (pH 7.0) and a peptide/lipid ratio 1:50, and were measured at 30 °C.

Many peptides have been reported to be α-helical in the presence of SDS; therefore, we wanted to obtain further experimental data concerning the importance of the choline head group of the phospholipid for helix formation by the peptide LytA_239–252_. To this end, we recorded far-UV CD spectra in two types of small unilamellar vesicles (SUVs): DMPC/DMPG (3:1) vesicles, formed by choline-phospholipids, and POPE/POPG (2:1) vesicles, formed by non-choline phospholipids. As seen in Figure 11, CD spectra of LytA_239–252_ in both types of vesicles exhibit a minimum at about 208 nm, and a shoulder at about 222 nm, which are characteristic of helical structures. The similarity between these spectra and that of DPC micelles (Figure 5 A) indicates that LytA_239–252_ forms the same helix structure in SUVs and in micelles.

More interestingly, the fact that the CD spectra in both types of vesicles are very similar indicates that the choline head group of the phospholipid is not the driving force for the transition from native-like β-hairpin to amphipathic α-helix.

### Effect of micelles on the structure of full-length C-LytA module

**Figure 12:**
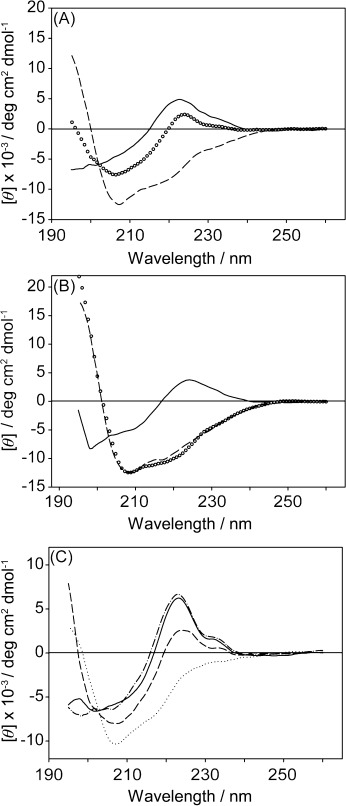
Effect of detergent micelles on the full-length C-LytA module: A) Experiments at pH 7.0 in the absence (solid line) and the presence of 30 mm DPC (circles) or 30 mm SDS (dashed line). B) Experiments at pH 3.0. Line scheme as above. C) Effect of temperature on DPC-induced C-LytA conformational changes. Experiments at pH 7.0 and 30 mm DPC: 5 °C (solid line), 25 °C (dashed line), 37 °C (dotted line) and 5 °C recorded 16 h after heating (dashed-dotted line).

The results shown so far demonstrate that a single CBR has the ability to interact with detergent micelles and undertake a dramatic conformational change. Nevertheless, CBRs are never found isolated in nature; they are arranged as linked units within the choline-binding modules, and usually display intramolecular interactions between them.^6^, 12b Therefore, we wanted to check whether the individual CBR propensities to become inserted into the micelles could be maintained in the framework of the full-length C-LytA module. As shown in Figure 12 A, at pH 7.0 and 25 °C, DPC micelles affect the far-UV CD spectrum of C-LytA, but SDS micelles clearly disrupt the anisotropic environment around the aromatic residues (loss of the positive band at 223 nm), while inducing an appreciable amount of α-helical structure (minimum at 208 nm and shoulder at 222 nm). At pH 3.0, where C-LytA is less stable,^31^ DPC micelles are able to complete the hairpin to helix transition to SDS levels (Figure 12 B). This suggests that insertion into the micelles requires some degree of flexibility in the protein to be accomplished and explains why SDS is more effective than DPC, because the former detergent is a strong denaturant that, in fact, has been described to fully unfold C-LytA at sub-micellar concentrations.6b To investigate this hypothesis, we analysed the effect of DPC micelles on C-LytA at pH 7.0 at different temperatures. As shown in Figure 12 C, the CD spectrum at 5 °C is similar to that in the absence of micelles (Figure 12 A), whereas, in contrast, at a physiological temperature in which C-LytA is more unstable (37 °C),6a, 12b a clear induction of α-helix can be seen, which is reversible upon cooling the sample. It can therefore be concluded that loosening the structure of the module either by temperature or pH greatly facilitates micelle insertion.

## Discussion

Anfinsen’s hypothesis postulates the existence of a univocal relationship between the protein sequence and its folded structure. The fact that nowadays it is possible to predict protein secondary structures quite successfully supports the conclusion that an unravelled protein folding code exits. However, one protein fold can be shared by quite unrelated sequences, and accurate folding rules have so far proven to be elusive. Furthermore, it is known that certain sequences, referred to as “chameleonic”,^32^ can be either helical or extended, depending on the context, usually depending on the rest of the protein in which they are located.^33^ In fact, because of its applicability in the fields of biosensors and biomaterials, there is growing interest in chameleonic peptides that are able to untertake a conformational change, if possible reversible, upon induction by controllable stimuli, such as pH,^34^ metal binding,^35^ and redox^36^ and photo-inducible switches.^37^ So far, reported conformational transitions in peptides correspond to: 1) random coil to ordered secondary structures,35b, 35c or to self-assembled hydrogel β-sheets;^34^ 2) soluble monomeric α-helix to self-associated oligomeric β-sheets;^36^ 3) different registers in coil-coiled helices,35a and 4) dimer of coil-coiled helices to helical-hairpin.^38^

Based on the spectroscopic studies described in this work, we demonstrate that a linear 14-residue peptide derived from the core of the third choline-binding repeat of the pneumococcal LytA autolysin, LytA_239–252_, forms a very stable native-like β-hairpin and has the ability to bind choline in aqueous solution. The peptide maintains the β-hairpin structure in the presence of TFE, but, unexpectedly, it converts into a stable α-helix in the presence of DPC or SDS micelles, as well as in DMPC/DMPG and POPE/POPG SUVs. This α-helix can fold back into the native-like β-hairpin by dilution to sub-micellar detergent concentration.

Random coil to α-helix transitions have been reported to be induced by methanol or fluorinated alcohols such as TFE and HFIP,^30^ or, in the case of antimicrobial peptides, by micelles.^39^ However, to our knowledge, LytA_239–252_ is the first documented case of a peptide that forms two completely different ordered structures depending on the solvent conditions. Moreover, reported chameleonic sequences are up to seven residues long in natural proteins,^40^ and even 11 in a particular designed sequence,^32^ so LytA_239–252_ represents the longest sequence known so far of this kind. Another interesting difference is that the LytA_239–252_ sequence can be predicted to form a β-hairpin by the program Betahairpred (http://triton.iqfr.csic.es/software/behairpredv1.0/behairpred.htm), but it is not predicted to be helical by AGADIR (http://agadir.crg.es), and only residues 240–246 show some very low helical propensity by PSIPRED (http://bioinf.cs.ucl.ac.uk/psipred/) (data not shown).

A clear-cut difference between the two structures formed by LytA_239–252_ is that whereas hydrophobic and polar side chains are evenly distributed between the two faces of the β-hairpin plane in aqueous solution, the α-helix is amphipathic, with hydrophobic residues clearly clustered in one face and polar/charged residues in the other (Figure 4 and 7). This amphipathic structure is very suitable to interact with a DPC micelle, as visualised in our model for the peptide/micelle complex (see the Supporting Information, Figure S7). In support, the side chains of the residues on the hydrophobic face are precisely those best defined in the NMR structure, probably as a consequence of their restricted mobility. In contrast, the nonamphipathic β-hairpin is not able to be inserted in the detergent micelles. The β-hairpins formed by the control peptides SESYV11 and SESYW11 are more amphipathic than their putative helical structure (see the Supporting Information, Figure S10), and hence more suitable to interact with micelles. This explanation is consistent with previous proposals concerning the importance of amphipathicity for the interaction with membranes of other peptides.^41^

Finally, it should be remarked that the conformational plasticity of peptides and proteins is at the basis of many relevant biological events. Well-known examples of these are the conformational helix to oligomer sheet transitions in the prion protein and the amyloid peptide, which cause important diseases. In *S. pneumoniae*, access of the pneumococcal LytA amidase and other CBPs to the cell wall from the cytosol implies the interaction with and translocation across the cell membrane without the use of a signal peptide. Our results suggest that CBRs, both individually and in the context of a full-length CBM, have the ability to undergo reversible disruption of their native structure and acquire an alternative, helical conformation with the ability to recognise the lipid bilayer; this might constitute a general mechanism to complete the sorting of these proteins to the bacterial surface to carry out their physiological activity.

## Conclusion

The structural behaviour of LytA_239–253_, a peptide encompassing the β-hairpin core of the third CBR of pneumococcal C-LytA, was exhaustively examined by means of CD, fluorescence and NMR analyses. We found that, as intended, the peptide forms a very stable native-like β-hairpin in aqueous solution, and also in the mixed trifluoroethanol/water solvent. Quite unexpectedly, the peptide structure becomes α-helical in the presence of zwitterionic and anionic detergent micelles, as well as in SUVs. That micelles are not general α-helix-inductors was confirmed by the fact that other unrelated peptides maintain their β-hairpin structures in micellar media. Based on the differences in the distribution of hydrophobic/polar side chains in theβ-hairpin and α-helix structures of LytA_239–252_, we propose that amphipathic structures are stabilised upon interaction with detergent micelles. The fact that many cationic antimicrobial and cell penetrating peptides are mainly disordered in aqueous solution and convert into amphipathic helices in micelles is consistent with our proposal. Accordingly, amphipathic β-hairpins remain stable in micelles, displaying no helical tendency, as occurs in peptides SESYW11 and SESYV11. To our knowledge, no other case of a micelle-induced transition between two stable ordered peptide structures has been reported. This finding is relevant in the field of peptide design. Furthermore, the reversibility of the observed β-hairpin to α-helix transition makes it of potential applicability for structure-switch biosensors. On the other hand, the “chameleonic” conformational behaviour of peptide LytA_239–252_ can play a role in the translocation of LytA to the pneumococcal surface.

## Experimental Section

### Materials

Dodecylphosphocholine (DPC), 1,2-dimyristoyl-*sn*-glycero-3- phosphocholine (DMPC), 1,2-dimyristoyl-*sn*-glycero-3-phospho- (1′-*rac*-glycerol) (sodium salt) (DMPG), 1-hexadecanoyl-2-(9*Z*-octadecenoyl)-*sn*-glycero-3-phosphoethanolamine (POPE) and 1-hexadecanoyl-2-(9*Z*-octadecenoyl)-*sn*-glycero-3-phospho-(1′-*rac*-glycerol) (sodium salt) (POPG) were purchased from Avanti Polar Lipids. Acrylamide, 1,6-diphenyl-1,3,5-hexatriene (DPH), sodium dodecyl phosphate (SDS), choline chloride, DEAE-cellulose, 5-doxyl-stearic acid (free radical) and methyl-16-doxyl-stearate (free radical) were from Sigma-Aldrich. The deuterated compounds [D_38_]DPC (98 %), [D_25_]SDS (98 %), [D_3_]TFE (99 %), [D_4_]MeOH (99.8 %), and D_2_O (99.9 %) were from Cambridge Isotope Laboratories (USA). The percentages of deuteration are indicated in parentheses.

### Peptide synthesis

Peptides LytA_239–252_, SESYV11 and SESYW11 were prepared in the solid phase by Fmoc (fluorenyl-9-methyloxycarbonyl) protocols and purified by reverse-phase HPLC up to 95 % or more purity by Caslo Aps (Lingbym, Denmark), DiverDrugs (Barcelona, Spain) and Lipotec (Barcelona, Spain), respectively.

**LytA_239–252_ (TGWKKIADKWYYFN)**: RP-HPLC: *t*_R_=10.9 min; 98.4 % (linear 18–36 % B gradient in 18 min; buffer A: 0.05 % TFA in H_2_O/CH_3_CN 98:2; buffer B: 0.05 % TFA in H_2_O/CH_3_CN 1:9). HRMS: Theoretical molecular weight: 1819.10; found: 1820.56 [*M* + H]^+^.

**SESYV11 (SESYINSDGTVTVTE)**: RP-HPLC: *t*_R_=14.7 min; 95.8 % (linear 15–25 % B gradient in 30 min; buffer A: 0.1 % TFA in H_2_O; buffer B: 0.07 % TFA in CH_3_CN). HRMS: Theoretical molecular weight: 1600.72; found: 1624.11 [*M* + Na]^+^.

**SESYW11 (SESYINSDGTWTVTE)**: RP-HPLC: *t*_R_=10.9 min; 95.5 % (21 % B in 20 min; buffer A: 0.1 % TFA in H_2_O; buffer B: 0.07 % TFA in CH_3_CN). HRMS: Theoretical molecular weight: 1687.72; found: 1688.85 [*M* + H]^+^.

### Protein purification

Wild-type C-LytA protein was purified by affinity chromatography from the overproducing *Escherichia coli* strain RB791 harbouring the pCE17 plasmid.12a Purified samples were subsequently dialyzed at 5 °C against 20 mm sodium phosphate buffer, pH 7.0, plus 50 mm NaCl, to remove the choline used for elution, and stored at −20 °C. Protein concentration was determined spectrophotometrically as described previously12a by using a molar extinction coefficient at 280 nm of 62 540 m^−1^ cm^−1^.

### Preparation of small unilamelar vesicles (SUVs)

For vesicle preparation, lipid powders (DMPC, DMPG, POPE and POPG) were dissolved in chloroform/methanol 50:50 (v/v) to obtain 5 mg mL^−1^ stock solutions. Aliquots of the stock solutions were mixed in glass vials and thoroughly vortexed to obtain DMPC/DMPG (3:1) and POPE/POPG (2:1) mixtures (molar ratios). Subsequently, the organic solvents were removed under a gentle stream of nitrogen, followed by overnight vacuum. The lipid film formed at the bottom of the vials was dispersed by addition of 10 mm phosphate buffer at pH 7.0, and 10 min of vigorous vortexing. The suspensions of multilamellar vesicles (MLVs) were then homogenised by 10 freeze-thaw cycles followed by 1 min vortexing after each cycle. Small unilamellar vesicles (SUVs) were then formed by sonication of MLVs for 16 min at 35 °C in a strong ultrasonic bath (UTR200, Hielscher, Germany).

### CD study

CD spectra were recorded with Jasco J-815 spectropolarimeters (Tokyo, Japan) equipped with either a Peltier PTC-423S system (samples in water and in detergent micelles) or a water-thermostatted rectangular cell holder (samples in vesicles). Peptide concentrations were determined from the 280 nm UV absorbance by using a molar extinction coefficient of 13 980 m^−1^ cm^−1^ for LytA_239–252._^42^

For samples in aqueous solution and in detergent micelles, the peptide concentration was 30 μm and the cuvette path lengths were 0.1 cm for far-UV region (250–195 nm) and 1.0 cm for near-UV region (320–250 nm). Samples were centrifuged 5 min prior to CD measuring, although no visible precipitate was seen. All measurements were carried out in triplicate at 5 and 25 °C in the presence of 20 mm glycine buffer at pH 3.0 or of 20 mm sodium phosphate buffer at pH 7.0. Isothermal wavelength spectra for these samples were acquired at a scan speed of 50 nm min^−1^ with a response time of 2 s and averaged over at least six scans.

Samples in vesicles were prepared by adding an aliquot of a 0.3 mm peptide stock solution in water to either DMPC/DMPG (3:1) or POPE/POPG (2:1) vesicle dispersion in 10 mm phosphate buffer at pH 7.0. The final peptide concentration was adjusted to obtain a peptide-to-lipid molar ratio (P/L) of 1:50, and it was around 28 μm. CD spectra for these samples were measured by using a quartz glass cell (Suprasil, Hellma, Mülheim, Germany) of 1 mm path length between 260 and 185 nm at 0.1 nm intervals. Spectra were recorded at 30 °C (i.e., above the phase-transition temperature of the lipids). Three repeat scans at a scan-rate of 10 nm min^−1^, 8 s response time and 1 nm bandwidth were averaged for each sample and for the baseline of the corresponding peptide-free sample.

After subtracting the baseline spectra from the sample spectra, CD data were processed with the adaptative smoothing method, which is part of the Jasco Spectra Analysis software. Molar ellipticities ([*θ*]) were expressed in unit of deg cm^2^ dmol^−1^, using the residue concentration of peptide.

For CD-monitored thermal denaturation experiments, the sample was layered with mineral oil to avoid evaporation, and the heating rate was 60 °C h^−1^. Thermal scans were fitted by least squares to the Gibbs–Helmholtz equation [Eq. (1)] in which Δ*G*^o^ (*T*) is the free energy of the transition at a temperature *T*, Δ*H*_m_ is the van’t Hoff enthalpy, *T*_m_ is the midpoint of denaturation (in Kelvin) and Δ*C_p_* is the difference in heat capacity between the native and denatured states.

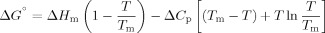
(1)

Stabilisation free energies (Δ*G*^o^) were calculated from the CD titration traces [Eq. (2)] in which *K*_eq_ is the equilibrium constant between the initial and final states, [*θ*]_I_ and [*θ*]_F_ are the ellipticities of the initial and final state, respectively, and [*θ*]_*x*_ is the experimental ellipticity at a given temperature.

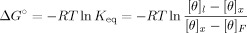
(2)

For choline titration, independent peptide samples were prepared in the presence of different ligand concentrations, and incubated for 5 min prior to recording the wavelength spectra. Binding was analysed according to a Langmuir analysis [Eq. 3)], considering only one binding site per peptide, and in which Δ[*θ*]_293_ is the change in ellipticity at 293 nm at each point, Δ[*θ*]_293_ (max) is the change in ellipticity at ligand saturation, and *K*_d_ is the dissociation constant.

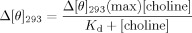
(3)

### NMR sample preparation

NMR samples were prepared by solving the lyophilised peptide (1–2 mg) in 0.5 mL of solvent; i.e., H_2_O/D_2_O (9:1 ratio by volume), pure D_2_O, 30 mm [D_38_]DPC in H_2_O/D_2_O (9:1 v/v), 30 mm [D_38_]DPC in D_2_O, 0.5 mm [D_38_]DPC in H_2_O/D_2_O (9:1 v/v), 0.5 mm [D_38_]DPC in D_2_O, 30 mm [D_25_]SDS in H_2_O/D_2_O (9:1 v/v), 30 mm [D_25_]SDS in D_2_O, 0.2 mm [D_25_]SDS in H_2_O/D_2_O (9:1 v/v), 0.2 mm [D_25_]SDS in D_2_O, 30 % [D_3_]TFE/70 % H_2_O/D_2_O 9:1, and 30 % [D_3_]TFE/70 % D_2_O. Peptide concentrations were 0.5–1.0 mm, except where another concentration is indicated. pH was adjusted to 3.0 by adding minimal amounts of NaOD or DCl, measured with a glass micro-electrode and not corrected for isotopic effects. Approximate peptide/detergent ratios are indicated in each case. Peptide and detergent were equimolar in the samples at sub-micellar detergent concentrations. All the samples were placed in 5 mm NMR tubes, and contained sodium 2,2-dimethyl-2-silapentane-5-sulfonate (DSS) as internal reference for ^1^H chemical shifts.

### NMR spectra acquisition

NMR spectra were recorded with a Bruker Avance-600 spectrometer operating at a proton frequency of 600.1 MHz and equipped with a cryoprobe, the temperature of which was calibrated by using a methanol sample. 1D ^1^H NMR spectra were acquired by using 32 K data points, which were zero-filled to 64 K data points prior to Fourier transformation. Phase-sensitive two-dimensional correlated spectroscopy (COSY), total correlated spectroscopy (TOCSY) and nuclear Overhauser enhancement spectroscopy (NOESY) spectra were recorded by standard techniques using the time-proportional phase increment mode. Water signal was suppressed by either presaturation or by using a 3–9–19 pulse sequence. TOCSY spectra were obtained by using 60 ms DIPSI2 with z filter spin-lock sequence. NOESY mixing time was 150 ms. ^1^H-^13^C heteronuclear single quantum coherence (HSQC) spectra were recorded at ^13^C natural abundance. Acquisition data matrices had 2048×512 points in *t*_2_ and *t*_1_, respectively. Data were processed with the standard TOPSPIN program (Bruker Biospin, Karlsruhe, Germany). The 2D data matrices were multiplied by a square-sine-bell window function with the corresponding shift optimised for every spectrum and zero-filled to 2×1 K complex matrices prior to Fourier transformation. Baseline correction was applied in both dimensions. ^13^C δ-values were indirectly referenced by using the IUPAC-IUB recommended ^1^H/^13^C chemical shift ratio (0.25144953).^43^

### NMR spectra assignment

^1^H NMR signals of peptide LytA_239–252_ in each solvent conditions and those of peptides SESYV11 and SESYW11 in [D_38_]DPC micelles were assigned by analyses of the 2D NMR spectra using the SPARKY software^49^ and the standard sequential assignment strategy.^44^ The ^13^C resonances were identified on the basis of the correlations between the protons and the bound carbon atoms present in the ^1^H,^13^C-HSQC spectra. These chemical shifts are listed in Tables S1 S3, and 4.

### Structure calculation

Structure calculation was done by following a two-step protocol. First, we applied the standard iterative procedure for automatic NOE assignment of the CYANA 2.1 program, which performs seven cycles of combined automated NOE assignment and structure calculation of 100 conformers per cycle.^45^ As experimental input data, we used the lists of: i) assigned chemical shifts (more than 99 % of ^1^H and ^13^C chemical shifts of all assignable nuclei were assigned in LytA_239–252_), ii) NOE integrated cross-peaks present in 150 ms NOESY spectra, and iii) φ and ψ dihedral angle restraints, which were derived from ^1^H and ^13^C chemical shifts using TALOS+ webserver.^46^ Integration of NOE cross-peaks was performed by the automatic integration subroutine of SPARKY software.^49^ For the structure of LytA_239–252_ in aqueous solution, we include NOE cross-peaks observed in two NOESY spectra acquired at 5 °C, one in H_2_O/D_2_O (9:1 ratio by volume) and the second in D_2_O. NOE cross-peaks for the structure in DPC and SDS micelles came from NOESY spectra recorded in 30 mm [D_38_]DPC at 35 °C, and in 30 mm [D_25_]SDS at 25 °C, respectively. The list of upper limit distance constraints resulting from the last automatic cycle was checked by inspection of the corresponding NOESY spectra, and ambiguous constraints were removed or relaxed to generate the final list used as input for a standard simulated annealing CYANA 2.1 calculation of 100 conformers. The final ensembles of the 20 lowest target function structures were visualised and examined by using the program MOLMOL,^22^ and their quality was assessed by using PROCHECK/NMR as implemented at the Protein Structure Validation Suite server (PSVS server: http://psvs-1 4-dev.nesg.org/).

### Fluorescence measurements

Fluorescence measurements were carried out at 25 °C with a PTI-QuantaMaster fluorimeter (Birmingham, NJ, USA), model QM-62003SE, using a 5×5 mm path-length cuvette and a peptide concentration of 1 μm. Buffer was 20 mm glycine buffer at pH 3.0. Tryptophan emission spectra were obtained by using an excitation wavelength of 280 nm, with excitation and emission slits of 1.0 nm and a scan rate of 60 nm min^−1^. The critical micelle concentration (cmc) of DPC in 20 mm glycine buffer at pH 3.0 and 25 °C was determined according to the procedure of Chattopadhyay and London (1984),^47^ using DPH as a fluorescence probe. The cuvette path length was 10×10 mm, and excitation and emission slits were set to 1 nm. Excitation wavelength was 360 nm.

For acrylamide quenching experiments, independent peptide samples at 30 μm were incubated for 5 min with different acrylamide concentrations in the presence or absence of 30 mm of DPC, and the wavelength spectrum was recorded. For each sample, a blank without peptide was subtracted from the recorded spectrum. Experiments were repeated at least three times. Data were analysed with the Stern–Volmer equation [Eq. (4)],^48^ in which *F*_0_ and *F* are the fluorescence intensities at 340 nm in the absence and presence of quencher, respectively, *K*_SV_ is the Stern–Volmer constant and [*Q*] is the quencher concentration.

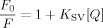
(4)

### Effect of paramagnetic compounds on NMR spectra

Samples of 0.5 mm LytA_239–252_ in 30 mm [D_38_]DPC in H_2_O/D_2_O 9:1 (v/v) pH 3.0 were titrated with three paramagnetic compounds: one hydro-soluble, MnCl_2_, and two liposoluble, 5-doxyl-stearic acid (free radical) and methyl-16-doxyl-stearate (free radical). Titrations were performed by adding aliquots (5–30 μL) from stock solutions of the paramagnetic agents, and monitored by 2D ^1^H,^1^H-TOCSY spectra acquired at 25 °C at each titration point. The stock solutions were 10–40 mm MnCl_2_ in H_2_O/D_2_O 9:1 (v/v) pH 3.0, 13 mm 5-doxyl-stearic acid in deuterated methanol ([D_4_]MeOH), and 12.6 mm methyl-16-doxyl-stearate in [D_4_]MeOH.
