# Supplementary material for: Micelle-Triggered β-Hairpin to α-Helix Transition in a 14-Residue Peptide from a Choline-Binding Repeat of the Pneumococcal Autolysin LytA
Source: Chemistry. 2015 Apr 27;21(22):8076–89. doi: 10.1002/chem.201500447 (PMC4471590; doi:10.1002/chem.201500447)
Supplement: Supplementary file 1 [file chem0021-8076-sd1.pdf]

CHEMISTRY
A European Journal

Supporting Information

Micelle-Triggered β-Hairpin to α-Helix Transition in a 14-Residue Peptide from a Choline-Binding Repeat of the Pneumococcal Autolysin LytA

Héctor Zamora-Carreras,[a] Beatriz Maestro,[b] Erik Strandberg,[c] Anne S. Ulrich,[c, d] Jesús M. Sanz,[b] and M. Ángeles Jiménez*[a]

chem_201500447_sm_miscellaneous_information.pdf

Supporting information

List of Figures:

Figure S1. Reversibility of the thermal unfolding of $LytA_{239-252}$ monitored by CD

Figure S2. ^{13}C profiles for $LytA_{239-252}$

Figure S3. $LytA_{239-252}$ interaction with DPC examined by CD

Figure S4. $^{1}H,^{13}C$-HSQC spectra showing the cross-peaks for the aromatic residues of $LytA_{239-252}$

Figure S5. NOE summary for $LytA_{239-252}$ in 30 mM DPC-d38

Figure S6. Effect of paramagnetic compounds on 2D $^{1}H,^{1}H$-TOCSY spectra of $LytA_{239-252}$ in 30 mM DPC-d38

Figure S7. Cartoon representation of a hypothetical model for $LytA_{239-252}$ helix/DPC micelles complex compatible with fluorescence and NMR data.

Figure S8. ^{13}C profiles for SESYW11 and SESYV11

Figure S9. Distribution of aromatic/hydrophobic and polar/charged side chains in the β-hairpin and in a putative helix for peptide SESYW11

Figure S10. Structure of $LytA_{239-252}$ in SDS micelles

List of Tables:

Table S1. ^{1}H and ^{13}C chemical shifts for $LytA_{239-252}$ in different solvent conditions at pH 3.0 and 25ºC

Table S2. Structural statistics parameters for the ensemble of the 20 lowest target function conformers calculated for peptide $LytA_{239-252}$ in aqueous solution and in micelles.

Table S3. ^{1}H and ^{13}C chemical shifts for peptide SESYV11 in 30 mM DPC

Table S4. ^{1}H and ^{13}C chemical shifts for peptide SESYV11 in 30 mM DPC

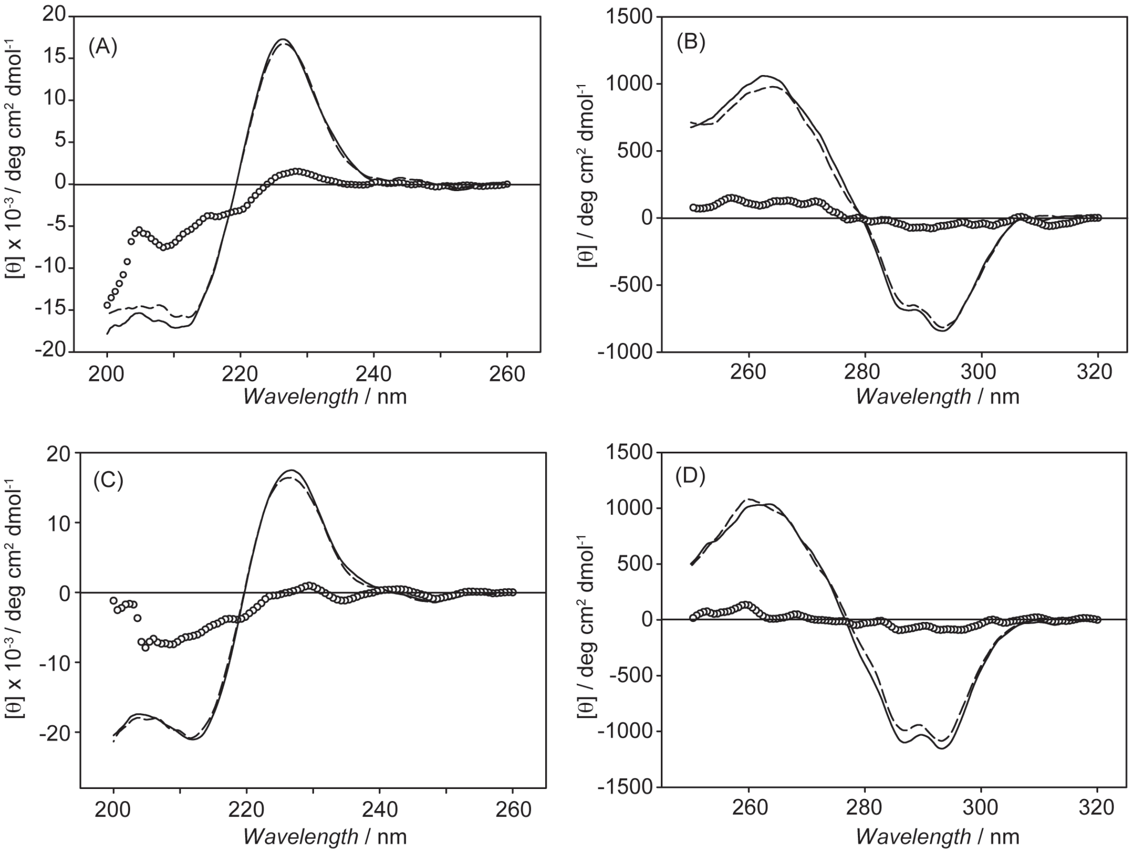

Figure S1. Reversibility of the thermal unfolding of LytA$_{239\text{-}252}$ monitored by CD. Panels A and B, far-UV and near-UV CD spectra, respectively, of LytA$_{239\text{-}252}$ recorded at 5 ºC prior to heating (solid line), 90º C (circles) and 5 ºC after heating (dashed line), in 20 mM glycine buffer, pH 3.0; Panels C and D, same as above but in the presence of 500 mM choline.

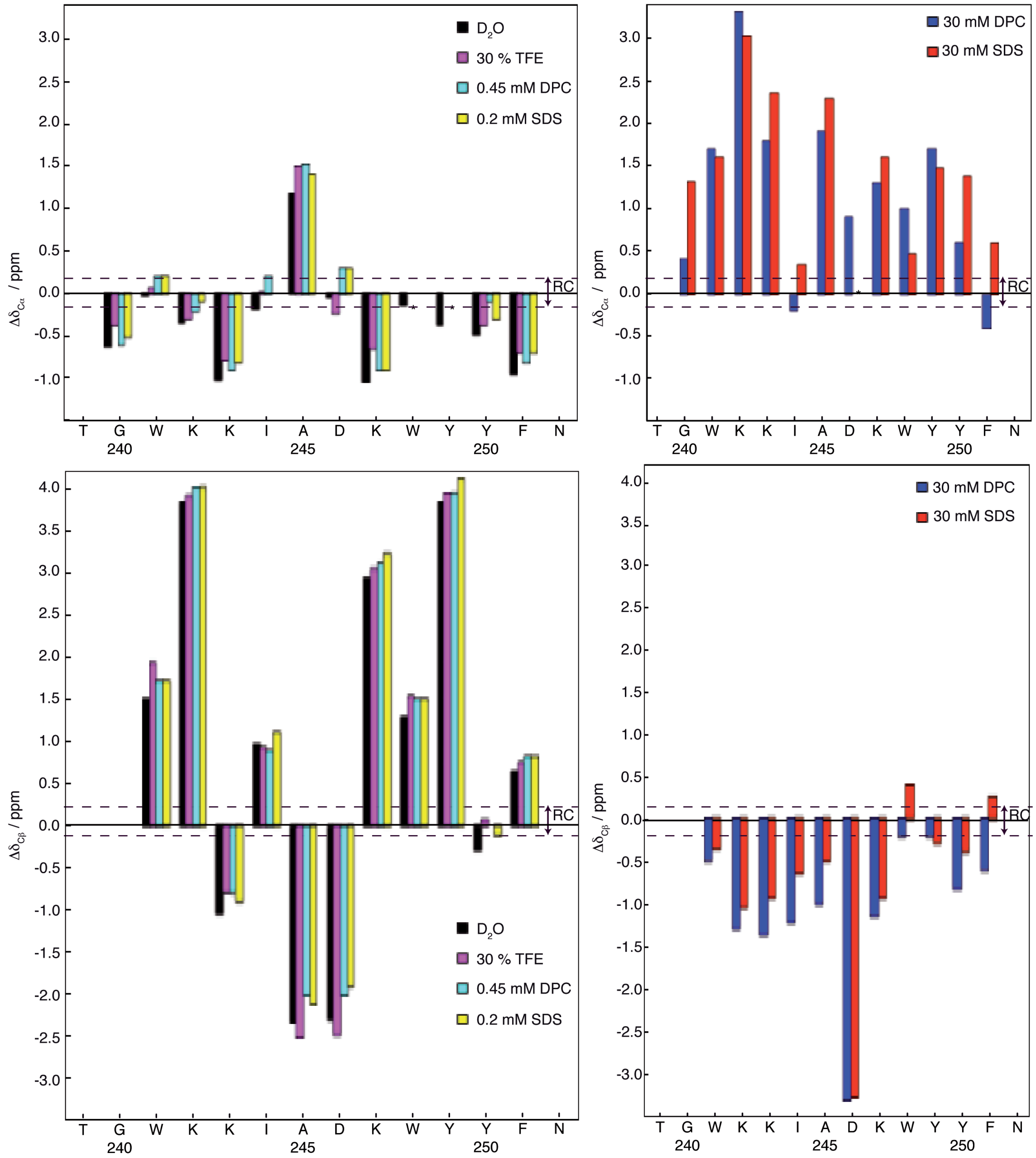

Figure S2. Bar plots of $\Delta\delta_{C\alpha}$ ($\Delta\delta_{C\alpha} = \delta_{C\alpha}^{observed} - \delta_{C\alpha}^{RC}$, ppm) and $\Delta\delta_{C\beta}$ ($\Delta\delta_{C\beta} = \delta_{C\beta}^{observed} - \delta_{C\beta}^{RC}$, ppm) as a function of peptide sequence for $LytA_{239\text{-}252}$ in different solvent conditions at pH 3.0 and 25 ºC. Bars colours for each solvent condition are indicated. $\delta_{C\alpha}^{RC}$ and $\delta_{C\beta}^{RC}$ values were taken from Wishart et al., *J. Biomol. NMR* 1995, 5, 67-81. The N- and C-terminal residues are not shown. The discontinuous lines indicate the random coil (RC) range, and the asterisks that the corresponding $\delta_{C\alpha}$ values were not determined.

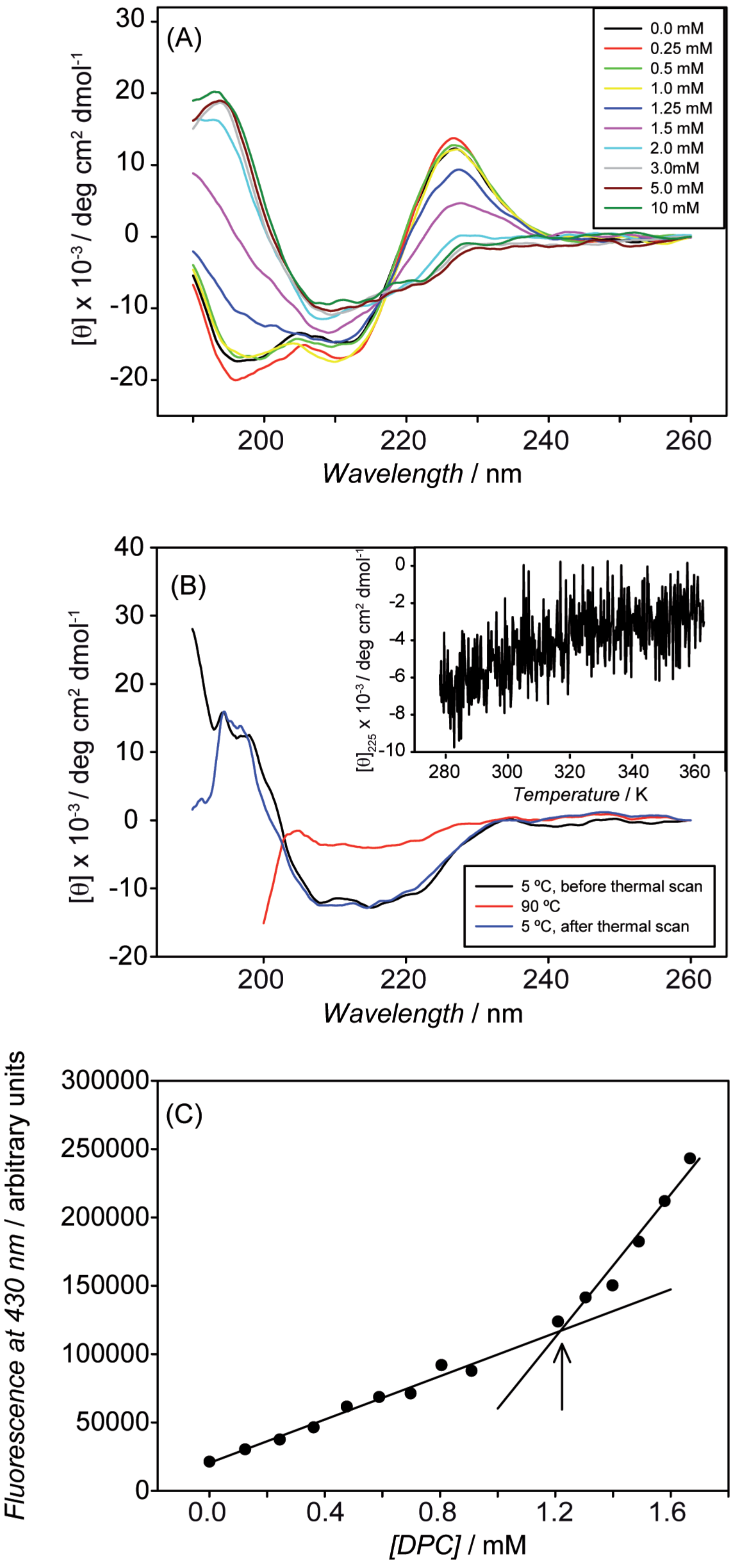

Figure S3. LytA$_{239-252}$ interaction with DPC examined by CD. (A) Far-UV CD-monitored titration of the peptide with DPC; (B) Thermal unfolding of LytA$_{239-252}$ in the presence of 30 mM DPC; far-UV CD spectra recorded at 5 ºC (black line), then at 90 ºC (red line) and finally at 5 ºC after cooling down the sample (blue line). *Inset*, temperature dependence of the ellipticity at 225 nm; (C) Determination of the critical micelle concentration of DPC in 20 mM Gly buffer, pH 3.0, using DPH as probe (see Materials and Methods). The arrow points to the determined cmc value.

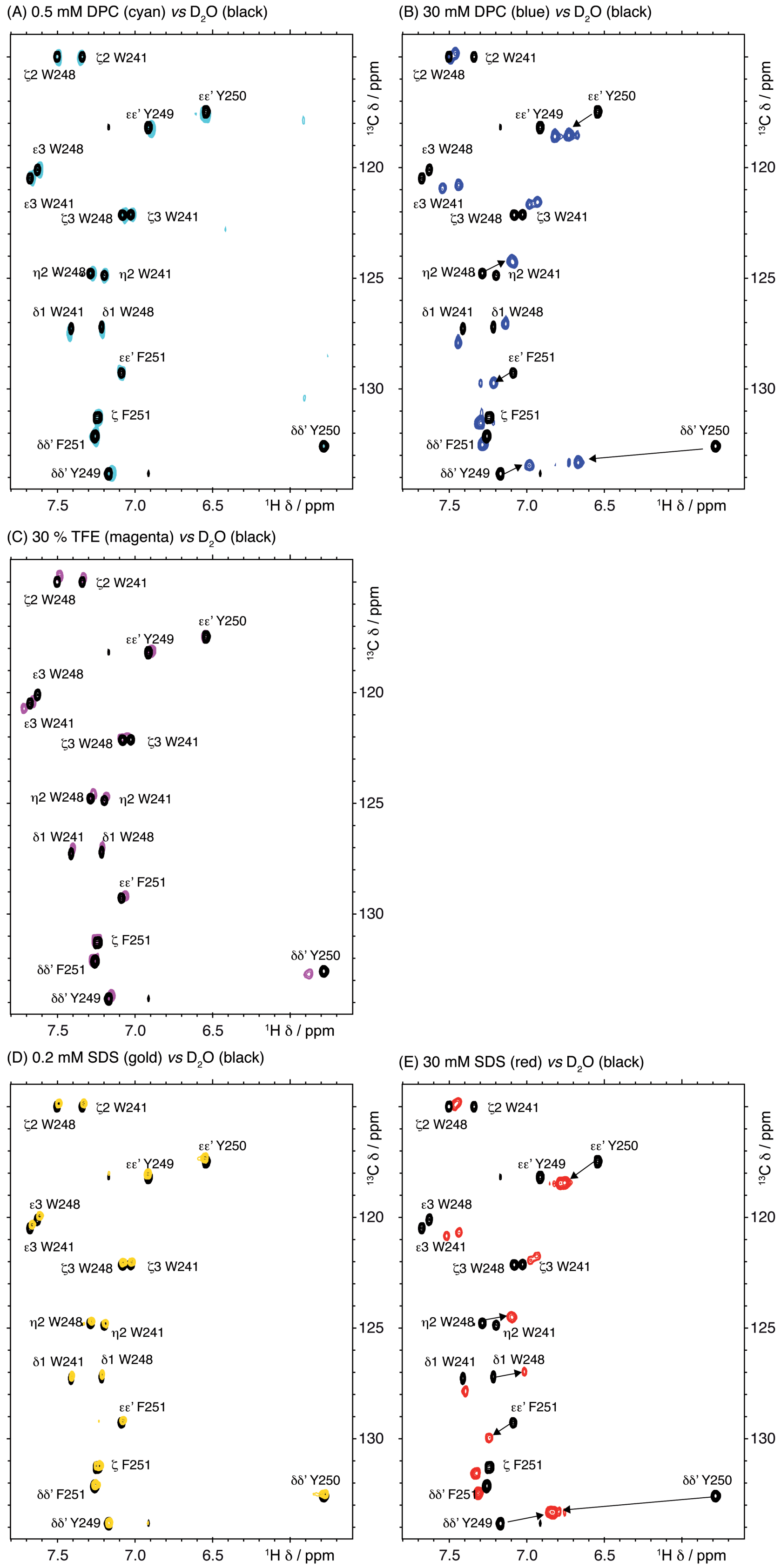

Figure S4. 2D $^{1}H,^{13}C$-HSQC spectral region showing the cross-peaks corresponding to the aromatic rings of Trp, Tyr and Phe residues recorded for $LytA_{239\text{-}252}$. (A) Spectra in D_2O (black contours) overlaid onto spectra in 0.5 mM DPC-d_{38} (cyan contours); (B) D_2O (black contours) onto 30 mM DPC-d_{38} (blue contours); (C) D_2O (black contours) onto 30% TFE (magenta contours); (D) 0.2 mM SDS-d_{25} (gold contours) onto D_2O (black contours); and (E) D_2O (black contours) onto 30 mM SDS-d_{25} (red contours). All at pH 3.0 and 25ºC.

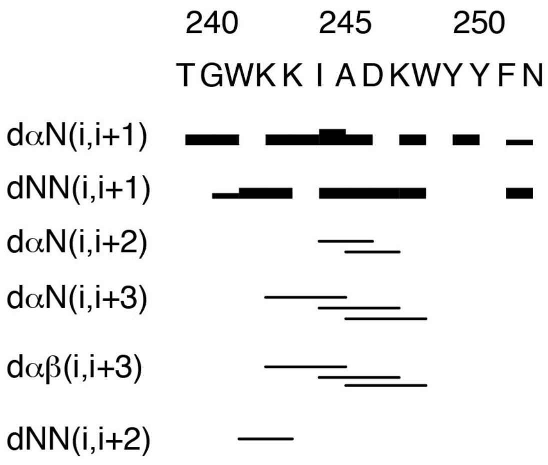

Figure S5. NOE summary for $LytA_{239\text{-}252}$ in 30 mM DPC-d_{38}, pH 3.0

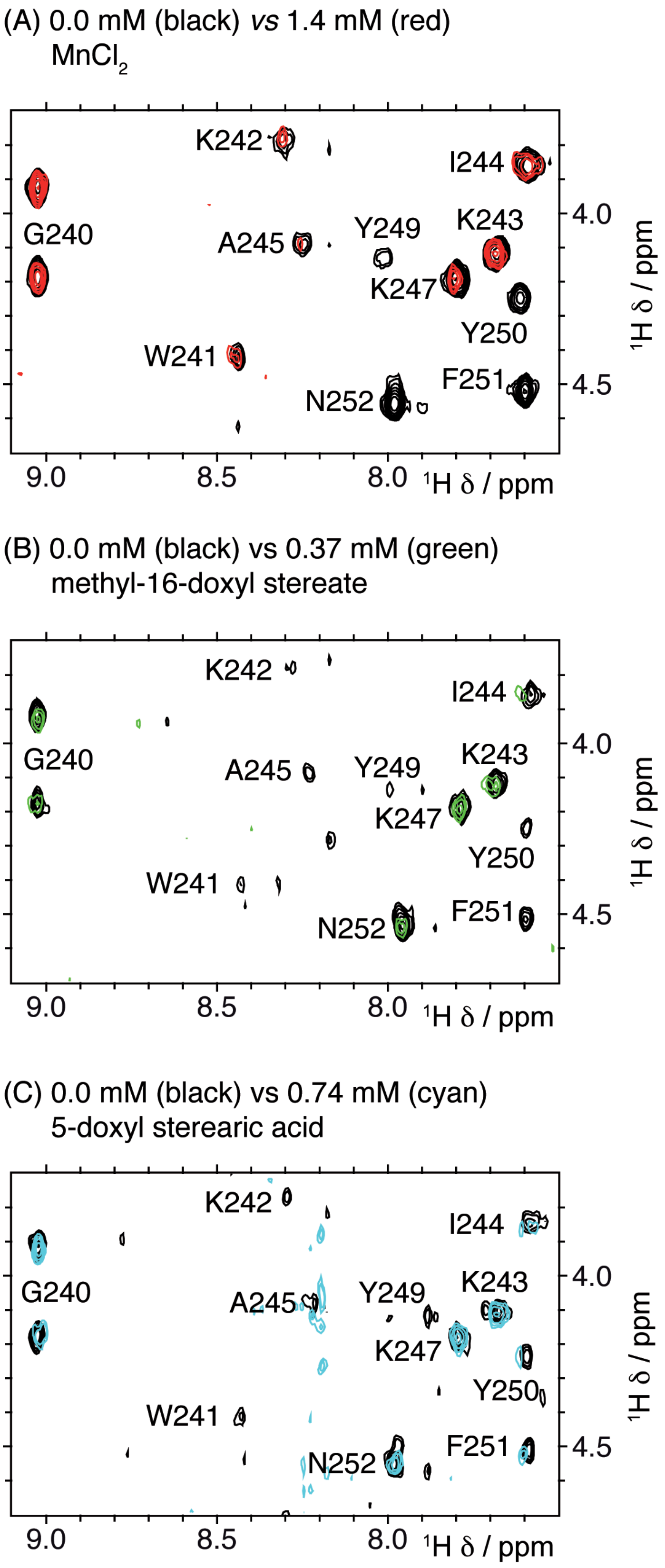

Figure S6. Effect of paramagnetic compounds in 2D ^{1}H,^{1}H-TOCSY spectra of 0.5 mM $LytA_{239-252}$ in 30 mM DPC-d_{38} in H_2O/D_2O 9:1 (v/v) pH 3.0 at 25 ºC. (A) $MnCl_2$ (B) methyl-16-doxyl stearate; and (C) 5-doxyl stearic acid.

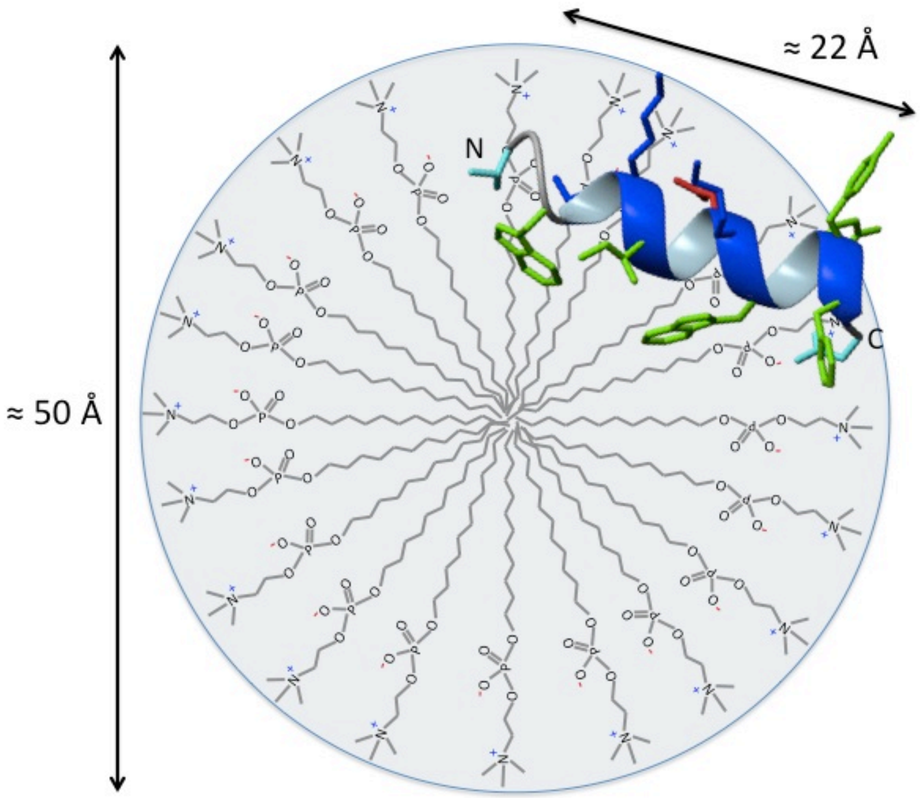

Figure S7. Cartoon representation of a hypothetical model for LytA$_{239\text{-}252}$ / DPC micelle complex. LytA$_{239\text{-}252}$ helix is displayed as a blue ribbon, and side chains in neon. Positively charged residues are in blue, negatively charged in red, Asn and Thr in cyan, and aromatic and hydrophobic in green. The amino and carboxylate termini are labelled by 'N' and 'C', respectively. The approximate lengths of the micelle diameter and LytA$_{239\text{-}252}$ helix are indicated.

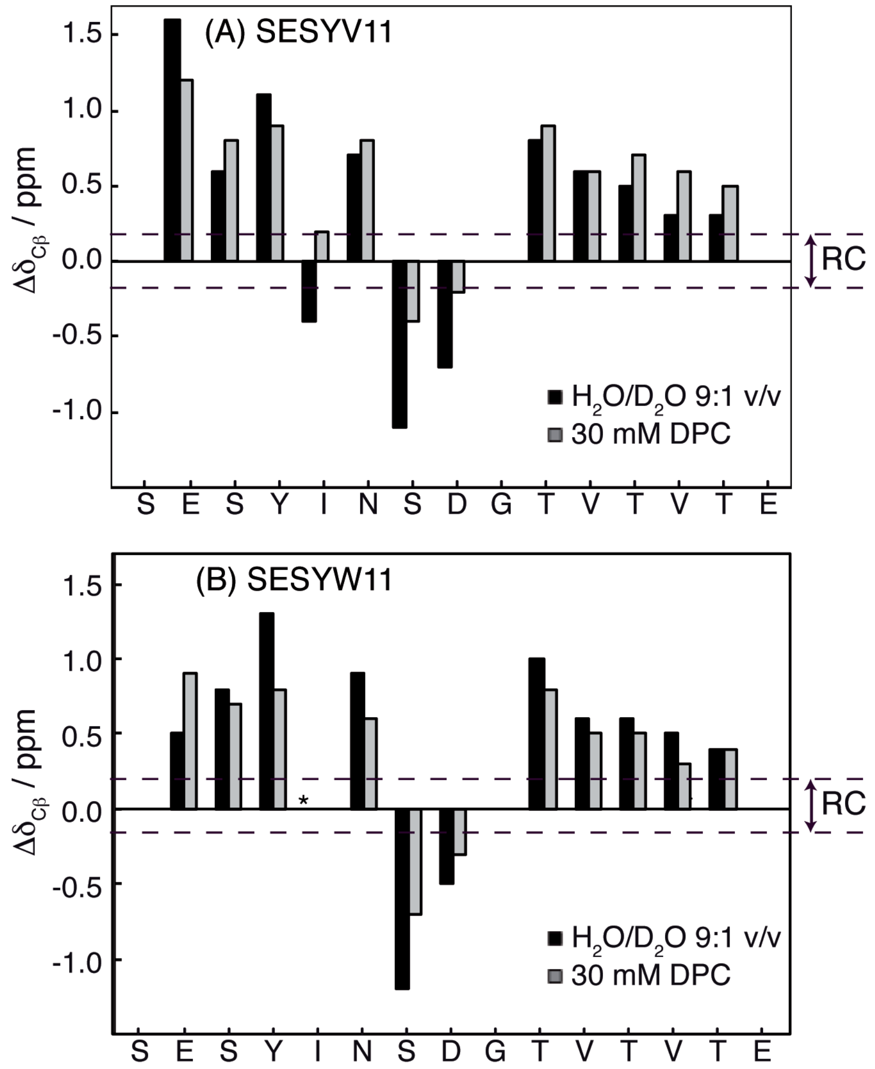

Figure S8. Bar plots of $\Delta\delta_{C\beta}$ ($\Delta\delta_{C\beta} = \delta_{C\beta}^{observed} - \delta_{C\beta}^{RC}$, ppm) as a function of peptide sequence for SESYW11 (A) and SESYV11 (B) in H_2O/D_2O 9:1 v/v at pH 5.5 and 5ºC (in black; data taken from Santiveri et al. *Protein Sci.* 2000 9, 2151-2160 & *J. Biomol. NMR* 2001, 19, 331-345), and in 30 mM DPC-d_{38} at pH 5.5 and 25ºC (in grey). $\delta_{C\beta}^{RC}$ values were taken from Wishart et al., *J. Biomol. NMR* 1995, 5, 67-81. The N- and C-terminal residues are not shown. The discontinuous lines indicate the random coil (RC) range, and the asterisks that the corresponding $\delta_{C\beta}$ values were not determined.

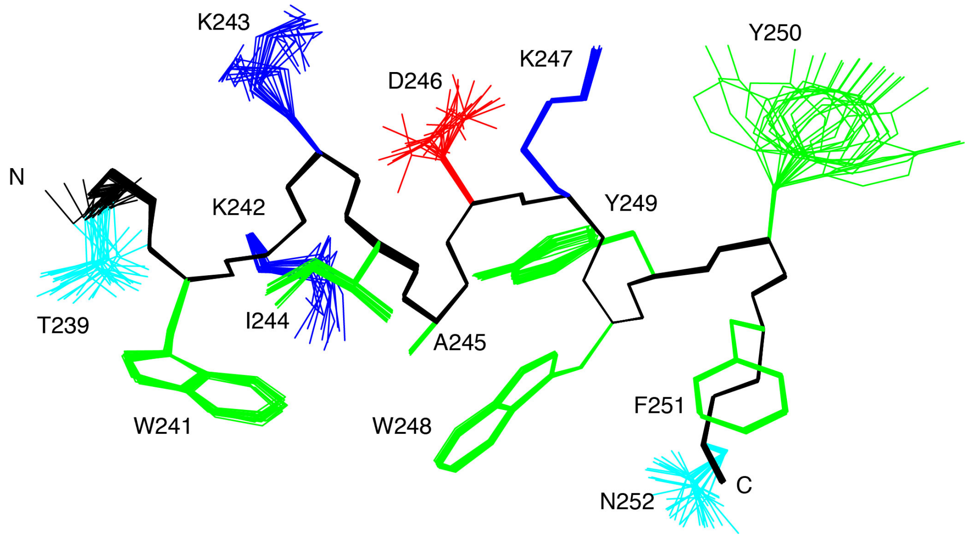

Figure S9. Structure of LytA$_{239\text{-}252}$ in SDS micelles. Ensemble of the 20 lowest target function structures overlaid onto the backbone atoms (black). Positively charged residues are in blue, negatively charged in red, Asn and Thr in cyan, and aromatic and hydrophobic in green. The amino and carboxylate termini are labelled by 'N' and 'C', respectively.

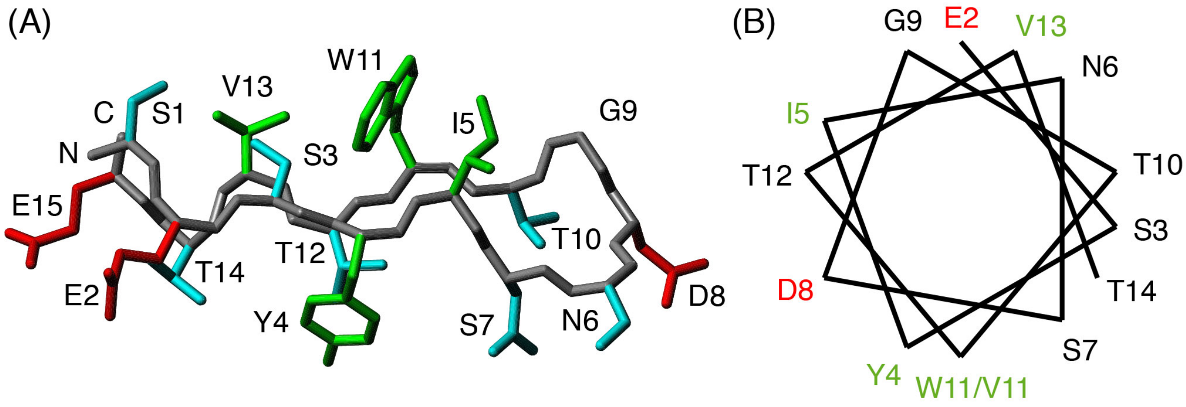

Figure S10. Distribution of aromatic/hydrophobic (green) and polar/charged (cyan/red) side chains in (A) the SESYW11 β-hairpin structure (Santiveri, Rico et al. 2000), and (B) a putative helix for peptides SESYW11 represented as a helical wheel. In panel A, the amino and carboxylate termini are labelled by 'N' and 'C', respectively.

Table S1. ^{1}H and ^{13}C chemical shifts (ppm from DSS) of $LytA_{239\text{-}252}$ under different solvent conditions at pH 3.0 and 25 ºC. "nd" stands for not determined. [a]Measured at 35ºC

	**Condition**	H_2O/D_2O 9:1 (v/v)	30 % TFE-d_3	0.45 mM DPC-d_{38},	30 mM DPC-d_{38}	30 mM SDS-d_{25}	0.2 mM SDS-d_{25}
**Peptide structure**		β-hairpin	β-hairpin	β-hairpin	α-helix	α-helix	β-hairpin
**Residue**	**Resonance**						
Thr 239	$C_\alpha H$	3.90	3.96	3.88	3.85	3.89	3.88
	$^{13}C_\alpha$	61.2	61.8	61.6	63.6	63.7	61.4
	$C_\beta H$	4.07	4.11	4.04	4.01	4.07	4.05
	$^{13}C_\beta$	69.5	69.7	69.8	68.5	69.1	69.8
	$C_\gamma H_3$	1.33	1.38	1.32	1.14	1.15	1.33
	$^{13}C_\gamma$	21.4	21.6	21.7	21.0	21.3	21.6
Gly 240	H_N	8.54	8.57	8.56	9.02	8.65	8.54
	$C_{\alpha\alpha'}H$	3.69, 4.48	3.73, 4.45	3.68, 4.47	3.92, 4.18	3.93, 4.07	3.66, 4.50
	$^{13}C_\alpha$	44.5	44.7	44.5	45.5	46.4	44.6
Trp 241	H_N	8.69	8.45	8.72	8.39	7.85	8.70
	$C_\alpha H$	5.45	5.51	5.43	4.47	4.46	5.44
	$^{13}C_\alpha$	57.5	57.6	57.7	59.2	59.1	57.7
	$C_\beta H$	3.19, 3.51	3.20, 3.56	3.19, 3.49	3.27, 3.29	3.32, 3.32	3.18, 3.50
	$^{13}C_\beta$	31.1	31.5	31.3	29.1[a]	29.2	31.3
	$C_{\delta1}H$	7.41	7.40	7.42	7.44	7.40	7.43
	$^{13}C_{\delta1}$	127.3	127.1	127.6	127.7	127.8	127.4
	$N_{\varepsilon1}H$	10.21	9.96	10.25	10.70	10.00	10.22
	$C_{\varepsilon3}H$	7.68	7.72	7.66	7.41	7.44	7.68
	$^{13}C_{\varepsilon3}$	120.5	120.8	120.7	120.7	120.6	120.6
	$C_{\zeta3}H$	7.03	7.05	7.02	6.91	6.94	7.04
	$^{13}C_{\zeta3}$	122.1	122.1	122.3	121.4	121.7	122.3
	$C_{\eta2}H$	7.20	7.18	7.19	7.07	7.09	7.21
	$^{13}C_{\eta2}$	124.9	124.7	125.1	124.1	124.4	125.1
	$C_{\zeta2}H$	7.34	7.33	7.35	7.48	7.48	7.35
	$^{13}C_{\zeta2}$	115.0	114.8	115.2	115.0	114.9	115.2
Lys 242	H_N	9.92	9.92	9.98	8.31	7.74	9.90
	$C_\alpha H$	4.86	4.86	4.86	3.82	3.84	4.86
	$^{13}C_\alpha$	55.9	55.9	56.0	59.5	59.2	56.1
	$C_{\beta\beta'}H$	1.90, 1.90	1.90, 1.94	1.85, 1.91	1.62, 1.73	1.60, 1.74	1.86, 1.92
	$^{13}C_\beta$	36.9	37.0	37.1	31.8[a]	32.1	37.1
	$C_{\gamma\gamma'}H$	1.43, 1.43	1.44, 1.44	1.41, 1.41	1.08, 1.14	1.07, 1.07	1.42, 1.42
	$^{13}C_\gamma$	25.0	25.1	25.2	24.8[a]	25.2	25.2
	$C_{\delta\delta'}H$	1.47, 1.54	1.53, 1.63	1.47, 1.53	1.62, 1.62	1.60, 1.60	1.46, 1.53
	$^{13}C_\delta$	29.2	29.0	29.3	28.7	29.2	29.3
	$C_{\varepsilon\varepsilon'}H$	2.74, 2.74	2.81, 2.81	2.74, 2.74	2.92, 2.92	2.94, 2.94	2.72, 2.72
	$^{13}C_\varepsilon$	41.7	42.0	41.9	41.6	42.3	41.9
	$N_\zeta H_3$	nd	nd	7.39	7.74	7.43	7.40
Lys 243	H_N	8.51	8.32	8.52	7.68	7.72	8.53
	$C_\alpha H$	4.47	4.53	4.45	4.11	4.11	4.46
	$^{13}C_\alpha$	55.2	55.4	55.3	58.0	58.6	55.4
	$C_{\beta\beta'}H$	-0.19, 1.18	-0.10, 1.25	-0.19, 1.18	1.83, 1.83	1.87, 1.87	-0.25, 1.17
	$^{13}C_\beta$	32.1	32.3	32.3	31.8[a]	32.2	32.2
	$C_{\gamma\gamma'}H$	-0.04, 0.60	0.08, 0.71	-0.05 0.60	1.42, 1.42	1.45, 1.50	-0.07, 0.58
	$^{13}C_\gamma$	24.5	24.7	24.8	24.5[a]	25.2	24.7
	$C_{\delta\delta'}H$	1.15, 1.15	1.20, 1.20	1.16, 1.16	1.69, 1.69	1.71, 1.71	1.13, 1.13
	$^{13}C_\delta$	29.1	29.3	29.4	28.5	29.2	29.3
	$C_{\varepsilon\varepsilon'}H$	2.50, 2.62	2.59, 2.69	2.49, 2.62	2.96, 2.96	2.95, 2.95	2.48, 2.61
	$^{13}C_\varepsilon$	41.5	41.8	41.9	41.4	42.3	41.6
	$N_\zeta H_3$	7.48	7.54	7.48	nd	7.43	7.48

	**Condition**	H_2O/D_2O 9:1 (v/v)	30 % TFE-d_3	0.45 mM DPC-d_{38},	30 mM DPC-d_{38}	30 mM SDS-d_{25}	0.2 mM SDS-d_{25}
	**Peptide structure**	β-hairpin	β-hairpin	β-hairpin	α-helix	α-helix	β-hairpin
**Residue**	**Resonance**						
Ile 244	H_N	9.06	9.04	9.09	7.60	7.62	9.07
	$C_\alpha H$	4.17	4.18	4.15	3.86	3.91	4.15
	$^{13}C_\alpha$	60.9	61.1	61.3	60.9	61.4	61.1
	$C_\beta H$	1.95	2.00	1.94	2.03	2.09	1.94
	$^{13}C_\beta$	39.7	39.7	39.9	37.6	38.2	39.9
	$C_{\gamma 2}H_3$	0.88	0.90	0.88	0.93	1.01	0.88
	$^{13}C_{\gamma 2}$	17	16.7	17.3	17.4	18.0	17.2
	$C_{\gamma\gamma 1'}H$	1.10, 1.50	1.12, 1.54	1.10, 1.50	1.20, 1.63	1.29, 1.68	1.10, 1.50
	$^{13}C_{\gamma 1}$	27.1	27.2	27.3	28.2	28.6	27.2
	$C_{\delta 1}H_3$	0.76	0.78	0.76	0.80	0.92	0.75
	$^{13}C_{\delta 1}$	12.9	12.5	13.2	12.9	13.4	13.1
Ala 245	H_N	9.20	8.97	9.23	8.26	8.07	9.23
	$C_\alpha H$	3.93	3.89	3.94	4.09	4.12	3.92
	$^{13}C_\alpha$	53.7	54.0	54.0	54.4	54.8	53.9
	$C_\beta H_3$	1.49	1.54	1.49	1.54	1.53	1.49
	$^{13}C_\beta$	16.8	16.6	17.1	18.1	18.6	17.0
Asp 246	H_N	8.52	8.47	8.53	8.44	8.21	8.52
	$C_\alpha H$	4.33	4.43	4.32	4.63	4.63	4.31
	$^{13}C_\alpha$	54.2	54.0	54.5	55.1	nd	54.5
	$C_{\beta\beta'}H$	2.92, 3.03	3.00, 3.00	2.91, 3.01	2.98, 2.98	2.97, 3.02	2.90, 3.00
	$^{13}C_\beta$	38.8	38.6	39.1	37.8	37.8	39.2
Lys 247	H_N	7.88	7.94	7.87	7.80	7.79	7.89
	$C_\alpha H$	4.62	4.63	4.60	4.19	4.17	4.61
	$^{13}C_\alpha$	55.2	55.5	55.3	57.5	57.8	55.3
	$C_{\beta\beta'}H$	1.75, 1.75	1.75, 1.84	1.72, 1.77	1.82, 1.90	1.74, 1.84	1.71, 1.76
	$^{13}C_\beta$	36.0	36.1	36.2	32.0[a]	32.2	36.3
	$C_{\gamma\gamma'}H$	1.27, 1.45	1.32, 1.50	1.26, 1.45	1.28, 1.42	1.27, 1.37	1.27, 1.46
	$^{13}C_\gamma$	25.2	25.3	25.4	24.4[a]	24.8	25.4
	$C_{\delta\delta'}H$	1.73, 1.73	1.77, 1.77	1.72, 1.72	1.62, 1.62	1.59, 1.59	1.71, 1.71
	$^{13}C_\delta$	29.0	29.1	29.3	28.5	28.9	29.1
	$C_{\varepsilon\varepsilon'}H$	2.99, 2.99	3.03, 3.03	2.99, 2.99	2.85, 2.85	2.90, 2.90	2.99, 2.99
	$^{13}C_\varepsilon$	42.1	42.4	42.5	41.4	42.4	42.3
	$N_\zeta H_3$	7.56	7.63	7.57	7.72	7.47	7.56
Trp 248	H_N	8.39	8.19	8.40	7.99	7.96	8.41
	$C_\alpha H$	5.07	5.13	5.04	4.50	4.57	5.06
	$^{13}C_\alpha$	57.4	57.5	57.5	58.5	58.0	57.5
	$C_{\beta\beta'}H$	2.99, 3.31	3.02, 3.37	3.01, 3.30	3.24, 3.36	3.26, 3.37	2.99, 3.31
	$^{13}C_\beta$	30.9	31.1	31.1	29.4[a]	30.0	31.1
	$C_{\delta 1}H$	7.22	7.21	7.21	7.14	7.03	7.21
	$^{13}C_{\delta 1}$	127.2	127.1	127.5	126.8	126.9	127.1
	$N_{\varepsilon 1}H$	10.11	9.96	10.13	10.51	9.89	10.12
	$C_{\varepsilon 3}H$	7.63	7.66	7.62	7.54	7.53	7.63
	$^{13}C_{\varepsilon 3}$	120.1	120.5	120.3	120.8	120.8	120.2
	$C_{\zeta 3}H$	7.08	7.09	7.07	6.97	6.99	7.10
	$^{13}C_{\zeta 3}$	122.1	122.1	122.4	121.6	121.9	122.3
	$C_{\eta 2}H$	7.29	7.26	7.27	7.08	7.11	7.31
	$^{13}C_{\eta 2}$	124.8	124.6	125.0	124.0	124.5	125.0
	$C_{\zeta 2}H$	7.50	7.49	7.50	7.46	7.46	7.51
	$^{13}C_{\zeta 2}$	115.0	114.8	115.2	114.8	114.8	115.2

	Condition	H_2O/D_2O 9:1 (v/v)	30 % TFE-d_3	0.45 mM DPC-d_{38},	30 mM DPC-d_{38}	30 mM SDS-d_{25}	0.2 mM SDS-d_{25}
	**Peptide structure**	β-hairpin	β-hairpin	β-hairpin	α-helix	α-helix	β-hairpin
**Residue**	**Resonance**						
Tyr 249	H_N	9.45	9.45	9.49	8.01	7.90	9.44
	$C_\alpha H$	4.81	nd	nd	4.12	4.23	nd
	$^{13}C_\alpha$	57.5	nd	nd	59.6	59.4	nd
	$C_{\beta\beta'}H$	2.77, 2.77	2.78, 2.84	2.75, 2.75	2.78, 2.90	2.89, 2.89	2.76, 2.76
	$^{13}C_\beta$	42.6	42.7	42.7	38.6[a]	38.5	42.9
	$C_{\delta\delta'}H$	7.16, 7.16	7.15, 7.15	7.15, 7.15	6.60, 6.60	6.86, 6.86	7.18, 7.18
	$^{13}C_\delta$	133.8	133.7	134.0	133.2	133.3	134.0
	$C_{\varepsilon\varepsilon'}H$	6.91, 6.91	6.90, 6.90	6.90, 6.90	6.70, 6.70	6.80, 6.80	6.92, 6.92
	$^{13}C_\varepsilon$	118.2	118.2	118.5	118.3	118.4	118.4
Tyr 250	H_N	8.46	8.17	8.48	7.62	7.47	8.48
	$C_\alpha H$	4.09	4.23	4.08	4.24	4.23	4.06
	$^{13}C_\alpha$	57.4	57.5	57.8	58.5	59.3	57.6
	$C_{\beta\beta'}H$	0.94, 2.18	1.16, 2.25	0.92, 2.19	2.70, 2.88	2.74, 2.81	0.87, 2.16
	$^{13}C_\beta$	38.5	38.8	38.8	38.0[a]	38.4	38.7
	$C_{\delta\delta'}H$	5.78, 5.78	5.87, 5.87	5.78, 5.78	7.02, 7.02	6.84, 6.84	5.75, 5.75
	$^{13}C_\delta$	132.6	132.8	132.8	133.4	133.3	132.7
	$C_{\varepsilon\varepsilon'}H$	6.54, 6.54	6.55, 6.55	6.55, 6.55	6.82, 6.82	6.77, 6.77	6.54, 6.54
	$^{13}C_\varepsilon$	117.5	117.6	117.8	118.4	118.4	117.6
Phe 251	H_N	7.77	7.93	7.76	7.61	7.62	7.79
	$C_\alpha H$	4.46	4.48	4.45	4.51	4.45	4.44
	$^{13}C_\alpha$	56.8	57.0	56.9	57.3	58.3	57.0
	$C_{\beta\beta'}H$	2.75, 3.01	2.78, 3.02	2.75, 3.01	3.03, 3.18	2.98, 3.16	2.73, 3.00
	$^{13}C_\beta$	40.3	40.3	40.4	39.0	39.9	40.4
	$C_{\delta\delta'}H$	7.26, 7.26	7.27, 7.27	7.25, 7.25	7.28, 7.28	7.32, 7.32	7.29, 7.29
	$^{13}C_\delta$	132.1	132.1	132.3	132.4	132.4	132.3
	$C_{\varepsilon\varepsilon'}H$	7.09, 7.09	7.06, 7.06	7.08, 7.08	7.20, 7.20	7.25, 7.25	7.10, 7.10
	$^{13}C_\varepsilon$	129.3	129.2	129.5	129.7	129.9	129.4
	$C_\zeta H$	7.25	7.24	7.24, 7.24	7.28	7.33	7.25
	$^{13}C_\zeta$	131.3	131.2	131.5	131.4	131.5	131.5
Asn 252	H_N	8.20	8.08	8.20	7.98	7.96	8.25
	$C_\alpha H$	4.38	4.45	4.37	4.56	4.58	4.34
	$^{13}C_\alpha$	54.5	54.4	54.9	52.1	51.3	55.0
	$C_{\beta\beta'}H$	2.68, 2.77	2.70, 2.80	2.67, 2.77	2.61, 2.71	2.43, 2.59	2.66, 2.77
	$^{13}C_\beta$	39.5	39.6	39.8	39.0	39.3	39.8
	$N_{\delta\delta'}H_2$	6.95, 7.58	6.86, 7.58	6.95, 7.60	6.75, 7.44	6.62, 7.15	6.97, 7.61

Table S2. Structural statistics parameters for the ensemble of the 20 lowest target function conformers calculated for peptide $LytA_{239-252}$ in aqueous solution and in micelles. [a] Close contacts are H atoms at distance <1.6 Å and heavy atoms at distance < 2.2 Å. [b] $\chi2$ ($\chi21$ in I244) range is also < 10º. [c] $\chi2$ ($\chi21$ in I244) range is < 30º. [d] $\chi3$ & $\chi4$ ranges are also < 10º.

	**Aqueous solution**	**DPC micelles**	**SDS micelles**
**Number of distance restraints**			
Intraresidue (i – j = 0)	39	75	93
Sequential (|i – j| = 1)	23	45	65
Medium range (1 < |i – j| < 5)	23	36	79
Long range (|i – j| ≥ 5)	87	0	0
Total number	172	156	237
Averaged total number per residue	12.3	11.1	16.9
**Number of dihedral angle constraints**			
Number of restricted ϕ angles	12	12	12
Number of restricted ψ angles	12	12	11
Total number	24	24	23
**Maximum violations per structure**			
Distance (Å)	0.12	0.00	0.00
Dihedral angle (º)	0.4	0.0	0.1
Number of close contacts [a]	0	0	0
**Deviations from ideal geometry**			
RMSD for Bond angles (º)	0.2	0.2	0.2
RMSD for Bond lengths (Å)	0.001	0.001	0.001
**Pairwise RMSD (Å)**			
Backbone atoms	0.3 ± 0.1	0.6 ± 0.2	0.2 ± 0.1
All heavy atoms	1.0 ± 0.1	1.4 ± 0.4	0.9 ± 0.2
**Residues with ordered side chains**			
$\chi1$ range < 10º	W241[b], K243[b], I244[b], K247, W248[b], Y249, Y250, F251	T239[b], W241[b], I244[b], K247[b], W248, Y249, F251	W241[b], K242[b], I244[c], K247[d], W248[b], Y249, F251
$\chi1$ range < 30º	K242[c], D246	K242, K243, Y250	K243, D246, N252
**Ramachandran plot (%)**			
Most favoured regions	72.7	100	90.9
Additionally allowed regions	27.3	0	9.1
Generously allowed regions	0	0	0
Disallowed regions	0	0	0

Table S3. ^{1}H and ^{13}C chemical shifts (ppm, from DSS) for peptide SESYW11 in 30 mM DPC-d38 in H_2O/D_2O 9:1 (v/v) at pH 5.5 and 25 ºC. “nd” stands for not determined.

Residue	HN	$^{13}C_\alpha$	$C_\alpha H$	$^{13}C_\beta$	$C_\beta H$	Others
Ser 1		57.4	4.18	63.1	3.93, 3.98	
Glu 2		56.1	4.48	30.8	1.90, 2.04	$C_\gamma H$ 2.27, 2.27; $^{13}C_\gamma$ 35.8
Ser 3	8.41	nd	4.43	64.5	3.75, 3.75	
Tyr 4	8.46	nd	4.72	39.6	2.89, 3.02	$C_\delta H$ 6.99, 6.99; $^{13}C_\delta$ 133.7<br>$C_\varepsilon H$ 6.75, 6.75; $^{13}C_\varepsilon$ 118.3
Ile 5	8.02	60.5	4.01	38.8	1.32	$C_{\gamma1}H$ 0.79, 0.96; $^{13}C_{\gamma1}$ 27.4<br>$C_{\gamma2}H_3$ 0.72; $^{13}C_{\gamma2}$ 16.9<br>$C_{\delta1}H_3$ 0.62; $^{13}C_{\delta1}$ 12.9
Asn 6	8.55	nd	nd	39.5	2.84, 2.84	$N_\gamma H_2$ 6.90, 7.42
Ser 7	8.66	60.1	4.26	63.1	3.85, 3.92	
Asp 8	8.01	nd	4.61	40.8	2.67, 2.85	
Gly 9	8.15	45.5	3.67, 4.09			
Thr 10	7.89	61.8	4.36	70.6	4.19	$C_\gamma H_3$ 1.12; $^{13}C_\gamma$ 21.6
Trp 11	8.46	nd	4.87	30.1	3.09, 3.18	$C_{\delta1}H$ 7.21; $^{13}C_{\delta1}$ 127.3; $N_{\varepsilon1}H$ 10.1;<br>$C_{\varepsilon3}H$ 7.47; $^{13}C_{\varepsilon3}$ 120.9<br>$C_{\zeta3}H$ 7.02; $^{13}C_{\zeta3}$ 122.0<br>$C_{\eta2}H$ 7.17; $^{13}C_{\eta2}$ 124.6<br>$C_{\zeta2}H$ 7.44; $^{13}C_{\zeta2}$ 114.9
Thr12	8.55	61.9	4.34	70.3	3.75	$C_\gamma H_3$ 1.07; $^{13}C_\gamma$ 21.5
Val 13	8.27	61.8	4.38	33.2	2.02	$C_\gamma H_3$ 0.91, 0.91; $^{13}C_\gamma$ 20.6, 21.2
Thr14	8.46	61.5	4.43	70.2	4.23	$C_\gamma H_3$ 1.20; $^{13}C_\gamma$ 21.5
Glu 15	8.11	58.0	4.14	30.8	1.89, 2.05	$C_\gamma H$ 2.24, 2.24; $^{13}C_\gamma$ 35.9

Table S4. ^{1}H and ^{13}C chemical shifts (ppm, from DSS) for peptide SESYV11 in 30 mM DPC-d38 in H_2O/D_2O 9:1 (v/v) at pH 5.5 and 25 ºC. "nd" stands for not determined.

Residue	HN	$^{13}C_\alpha$	$C_\alpha H$	$^{13}C_\beta$	$C_\beta H$	Others
Ser 1		57.6	4.19	63.4	3.92, 3.98	
Glu 2	8.78	nd	4.46	31.1	1.88, 2.00	$C_\gamma H$ 2.25, 2.25; $^{13}C_\gamma$ 36.0
Ser 3	8.40	nd	4.70	64.6	3.71, 3.71	
Tyr 4	8.43	nd	4.66	39.7	2.91, 3.01	$C_\delta H$ 7.03, 7.03; $C_\varepsilon H$ 6.78, 6.78
Ile 5	8.09	60.9	4.23	39.0	1.72	$C_{\gamma1}H$ 1.07, 1.42; $^{13}C_{\gamma1}$ 27.6<br>$C_{\gamma2}H_3$ 0.86; $^{13}C_{\gamma2}$ 17.5<br>$C_{\delta1}H_3$ 0.79; $^{13}C_{\delta1}$ 13.1
Asn 6	8.64	nd	4.61	39.7	2.88, 2.88	$N_\gamma H_2$ 7.03, 7.48
Ser 7	8.68	60.1	4.31	63.4	3.87, 3.95	
Asp 8	8.08	nd	4.61	40.9	2.70, 2.86	
Gly 9	8.24	45.7	3.76, 4.13			
Thr 10	8.03	62.4	4.33	70.7	4.20	$C_\gamma H_3$ 1.16; $^{13}C_\gamma$ 21.9
Val 11	8.32	61.7	4.41	33.5	1.98	$C_\gamma H_3$ 0.86, 0.91; $^{13}C_\gamma$ 21.4, 20.3
Thr12	8.66	62.2	4.39	70.5	3.89	$C_\gamma H_3$ 1.14; $^{13}C_\gamma$ 22.0
Val 13	8.42	62.2	4.39	33.5	2.03	$C_\gamma H_3$ 0.90, 0.90; $^{13}C_\gamma$ 20.9, 20.9
Thr14	8.48	61.6	4.42	70.3	4.21	$C_\gamma H_3$ 1.18; $^{13}C_\gamma$ 21.7
Glu 15	8.11	58.2	4.13	31.1	1.89, 2.05	$C_\gamma H$ 2.23, 2.23; $^{13}C_\gamma$ 36.2
